# Single paternal dexamethasone challenge programs offspring metabolism and reveals multiple candidates in RNA-mediated inheritance

**DOI:** 10.1016/j.isci.2021.102870

**Published:** 2021-07-16

**Authors:** Katharina Gapp, Guillermo E. Parada, Fridolin Gross, Alberto Corcoba, Jasmine Kaur, Evelyn Grau, Martin Hemberg, Johannes Bohacek, Eric A. Miska

**Affiliations:** 1 Gurdon Institute, University of Cambridge, Cambridge, CB2 1QN, UK; 2 Wellcome Sanger Institute, Hinxton, CB10 1SA, UK; 3 Laboratory of Molecular and Behavioral Neuroscience, Institute for Neuroscience, Department of Health Sciences and Technology, ETH Zürich, Zürich, 8057, Switzerland; 4 Neuroscience Center Zurich, ETH Zurich and University of Zurich, Zürich, 8057, Switzerland; 5 Department of Genetics, University of Cambridge, Cambridge, CB2 3EH, UK; 6 Evergrande Center for Immunologic Diseases, Harvard Medical School and Brigham and Women's Hospital, Boston, MA 02215, USA; 7 Department of Medicine, CITIID, University of Cambridge, Cambridge CB2 0AW, UK

**Keywords:** Molecular physiology, Developmental biology, Embryology, Transcriptomics

## Abstract

Single traumatic events that elicit an exaggerated stress response can lead to the development of neuropsychiatric conditions. Rodent studies suggested germline RNA as a mediator of effects of chronic environmental exposures to the progeny. The effects of an acute paternal stress exposure on the germline and their potential consequences on offspring remain to be seen. We find that acute administration of an agonist for the stress-sensitive Glucocorticoid receptor, using the common corticosteroid dexamethasone, affects the RNA payload of mature sperm as soon as 3 hr after exposure. It further impacts early embryonic transcriptional trajectories, as determined by single-embryo sequencing, and metabolism in the offspring.

We show persistent regulation of tRNA fragments in sperm and descendant 2-cell embryos, suggesting transmission from sperm to embryo. Lastly, we unravel environmentally induced alterations in sperm circRNAs and their targets in the early embryo, highlighting this class as an additional candidate in RNA-mediated inheritance of disease risk.

## Highlights

• Single dexamethasone injection has consequences on offspring phenotype
• Dexamethasone induces dynamic changes in caput and cauda sperm and serum
• Dexamethasone induces changes in circRNAs in mature sperm

## Graphical abstract

**Figure:**
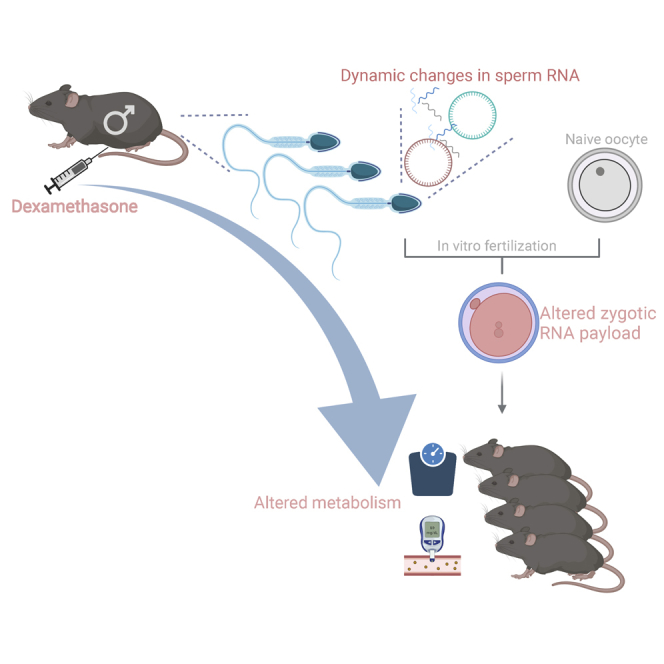

## Introduction

Acute stress elicits a complex but well-studied cascade of neuroendocrine responses regulated by the hypothalamic-pituitary-adrenal axis. It involves the release of neuropeptides in the brain that induce the secretion of corticosteroid hormones from the adrenals. These hormones in turn activate two types of corticosteroid receptors, glucocorticoid receptors (GRs) and mineralocorticoid receptor (MRs). These receptors are widely expressed throughout the body and regulate gene expression, thus enabling physiological and behavioral adjustments in response to stress (de Kloet et al., 2005). In vulnerable individuals, this response is excessive and it can lead to long-lasting maladaptive changes with consequences for psychological and metabolic health (Daskalakis et al., 2012).

It is also known that parental experiences can compromise the health of their progeny both in humans (Pembrey et al., 2006; Heijmans et al., 2008; Bowers and Yehuda, 2016) and in animal models (Benyshek et al., 2006; Roth et al., 2009; Jimenez-Chillaron et al., 2009; Carone et al., 2010; Pentinat et al., 2010; Shankar et al., 2010; Franklin et al., 2010; 2011; Morgan and Bale, 2011; Weiss et al., 2011; Dietz et al., 2011; Vassoler et al., 2013; Fullston et al., 2013; Martínez et al., 2014; Gapp et al., 2014; Marco et al., 2014; Rodgers et al., 2015; Sharma et al., 2015; Wu et al., 2016; Chen et al., 2016a; 2016b; Y. Y. Zhang et al., 2018). Research on the underlying mechanism of such transmission has found changes in germline epigenetic makeup, in particular DNA methylation, histone posttranslational modifications (PTMs), histone positioning, and RNA (Gapp and Bohacek, 2017). These epigenetic regulators are responsive to the environment and have been implicated in a variety of environmentally induced diseases (Jirtle and Skinner, 2007). Altered modifications must circumvent epigenetic reprogramming events in zygote and, depending on the timing of exposure, during germline development (Bohacek and Mansuy, 2017; Gapp and Bohacek, 2017). In the male germline, RNA is excluded from reprogramming and therefore a promising candidate for transgenerational information delivery (Gapp and Bohacek, 2017; Bohacek and Rassoulzadegan, 2019). Several studies carried out in *Drosophila* *melanogaster* and *Caenorhabditis* *elegans* reported on transgenerational inheritance of induced traits and provided firm evidence for the involvement of small RNAs in the mechanism of transmission (Ashe et al., 2012; Grentzinger et al., 2012; Shirayama et al., 2012). In mammals, a causal implication in the transmission of environmentally induced effects across generations has been demonstrated for sperm RNA only (Gapp et al., 2014; Grandjean et al., 2015; Sharma et al., 2015; Chen et al., 2016a, 2016b). Such RNA differs substantially from somatic RNA because it mainly consists of small RNA, predominantly tRNA-derived small fragments (tsRNAs), but also miRNAs, piRNAs, and circRNAs, among others (Chen et al., 2016b; Gapp and Bohacek, 2017; Bohacek and Rassoulzadegan, 2019). circRNAs comprise a very stable class of RNA that has recently been observed to be present in high amounts in testes but also to some extent in sperm (Barrett and Salzman, 2016). Some have been shown to act as miRNA sponges, thereby competing with mRNA targets while also regulating the expression of their host genes (Barrett and Salzman, 2016). Hence, circRNAs have a strong potential for amplifying an inherited signal, which makes them exceptionally interesting candidates for epigenetic germline inheritance. To date, the involvement of circRNAs in soma-to-germline signaling has not yet been investigated.

tsRNAs and miRNAs are crucial regulators of early embryonic development and players in nongenetic inheritance (Gapp et al., 2014; Grandjean et al., 2015; Rodgers et al., 2015; Sharma et al., 2015; Chen et al., 2016a, 2016b; Benito et al., 2018; Tyebji et al., 2020). They have been reported to be acquired through exosomal uptake during epididymal transfer from caput to cauda epididymis (Sharma et al., 2015, Sharma et al., 2018). This might explain their responsiveness to environmental perturbations, despite mature sperm's presumably transcriptionally silent state caused by tightly packed chromatin. Sperm RNA can indeed change in response to chronic stress or by chronic treatments that mimic stress exposure, such as repeated injection of GR agonists (Rodgers et al., 2013; Gapp et al., 2014; Short et al., 2016; Wu et al., 2016). In mice, uptake of epididymosomal miRNA was sufficient to replicate a chronic-stress-induced effect on stress response in the offspring (Chan et al., 2020). Surprisingly, acute stress has also recently been shown to affect offspring weight and glucose metabolism in mice (Hoyer et al., 2013) and some of these effects were germline dependent (Bohacek et al., 2016). Together, these related lines of evidence led us to hypothesize that acute GR activation has an intergenerational effect on offspring phenotype and that the transmission potentially implicates changes in the germline. The male germline cells – including mature sperm (Kaufmann et al., 1992; Haeussler and Claus, 2007) and their surrounding Sertoli cells (Hazra et al., 2014) – as well as the epididymal epithelial cells (Silva et al., 2010) express GRs that mediate the effects of glucocorticoids on transcription. Dexamethasone (Dex), a specific GR agonist, is known to directly activate GR in the rat epididymis (Silva et al., 2014). It is unknown whether acute stress affects sperm RNA, and if so, whether uptake via epididymosomes is involved in establishing germline changes that are relevant for offspring phenotypic alterations.

Here we investigate the impact of acute GR agonist administration on the germline RNA payload including circRNAs, at various time points after administration and interrogate the fate of altered sperm RNA. We further test germline dependency of transmitted metabolic effects and dissect the underlying molecular trajectories during early embryonic development using single-cell sequencing of embryos derived via *in vitro* fertilization (IVF). Identifying a readout of transgenerational risk load at the level of the paternal sperm epigenome could pave the way for future studies aiming at a prevention of the transmission of the effects of acute GR activation to the offspring.

## Results

### Effects of acute Dex injection on the germline small RNA payload

**Figure 1:**
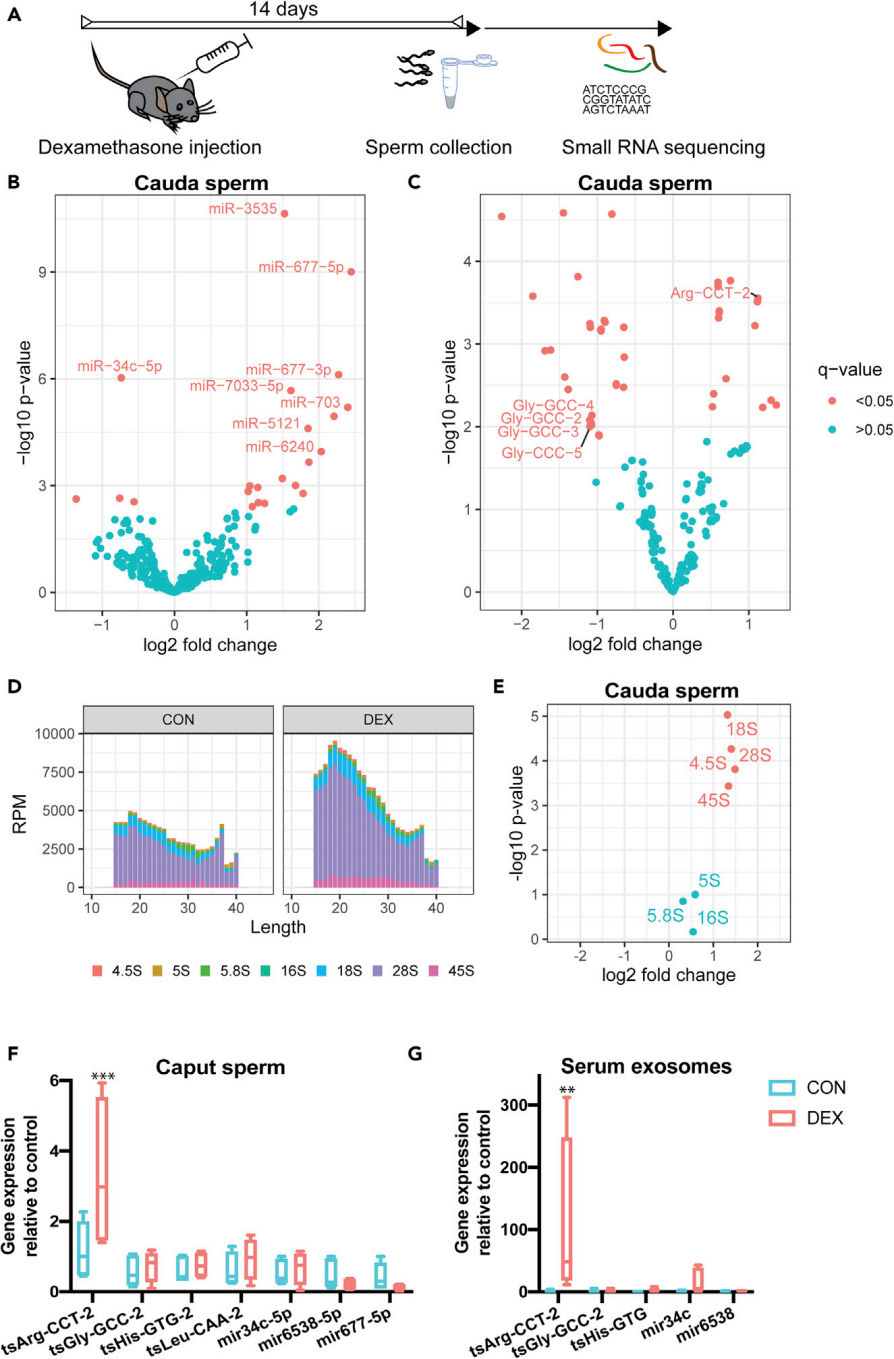
Effects of Dex on small RNA payload of sperm cells residing in testes at the time of administration (A) Experimental design depicting a time window of two weeks between injection of Dex and sperm collection for molecular analysis. (B and C) (B) Volcano plot depicting fold changes and significance level of miRNAs and tsRNAs (C) in mature sperm 14 days after injection of Dex (n = 8) versus vehicle (n = 8) as assessed by small RNA sequencing. (D) Stacked barplot showing reads of different sizes mapping to rRNAs in Dex (n = 8) and vehicle (n = 8). (E) Volcano plot demonstrating global increased abundance of rRNA fragments. (F and G) (F) Q-PCR results of small RNA assays for caput sperm (tsArg-CCT-2: t(49) = 3.49, q < 0.001, tsGlyGCC: t(49) = 0,32, q > 0.05; tsHIs-GTG: t(49) = 0.29, q > 0.05; tsLeu-CAA-2: t(49) = 0.62, q < 0.5; mir34c-5p: t(49) = 0.27, q > 0.05; mir6538: t(49) = 0.59, q > 0.05, mir677-5p t(49) = 0.62 q > 0.05) (G) serum exosomes (tsArg-CCT-2: t(33) = 3.43, q < 0.01, tsGlyGCC: t(33) = 0,04, q > 0.05; tsHIs-GTG: t(33) = 0.07, q > 0.05; mir34c-5p: t(33) = 0.51, q > 0.05; mir6538: t(33) = 0.02, q > 0.05) days after drug injection. Whiskers display minimum and maximum. ∗∗q < 0.01, ∗∗∗q < 0.001 multiple t-tests corrected for multiple testing.

Two reports have suggested that a single foot shock could elicit effects on offspring phenotype (Hoyer et al., 2013; Bohacek et al., 2016). To examine potential epigenetic mediators of such acute stressful impacts, we investigated sperm RNA of males 2 weeks after a single activation of the GR (Figure 1A). This timeline was chosen to mimic the timing at which breeding occurred when effects on offspring had been observed in a previous study (Bohacek et al., 2016). We injected the specific GR agonist Dex once intraperitoneally into 8 adult males. This drug is in frequent clinical use, now also as an apparently effective treatment for patients suffering from lower respiratory tract infection as a consequence of COVID-19 virus (EU Clinical Trials Register, n.d.; Biggest COVID-19 trial tests repurposed drugs first, 2020; Horby et al., 2020). A sperm population was harvested from each animal, and RNA was extracted for ultradeep small RNA sequencing, resulting in 16 libraries representing one injected male each (8 vehicle and 8 Dex-injected). Purity of the sperm samples was confirmed by inspecting RNA size profiles generated on the bioanalyzer to be absent of ribosomal RNA peaks, which would indicate contamination by somatic cells. Reaching an average of 55.4 million sequencing reads while also using randomized adapters for 3′ ligation put us in a position to reduce polymerase chain reaction (PCR) biases (Dard-Dascot et al., 2018) and accurately quantify less abundant miRNAs that are by far outnumbered in sperm by other small RNAs, e.g., tsRNAs(Peng et al., 2012). Our data showed an average of 60% mappable reads across all libraries, including 34% of multimappers. We detected an expected dominant prevalence of reads mapping to tsRNAs and abundant miRNAs in all samples (Figure S1A). Differential gene expression analysis, using DEseq2 (Love et al., 2014), revealed that a single acute activation of GR receptors induced changes in tsRNAs, miRNAs, and rRNAs collected 14 days after injection (Figures 1B–1E, FDR q < 0.05, Tables 1 and 2), as has been observed in response to chronic environmental stress previously (Rodgers et al., 2013; Gapp et al., 2014). Interestingly, tsRNA-Gly-GCC, a tsRNA previously associated with the effects of nutritional challenge (Sharma et al., 2015), was among the most strongly altered tsRNAs. We further detected changes in ribosomal-RNA-derived RNA (rsRNA) as has been observed in studies investigating the impact of high-fat and high-sugar diet (Y. Zhang et al., 2018; Nätt et al., 2019) (Figures 1D and 1E). It should be noted that our analysis only detects relative changes within the total small RNA pool. Thus, we cannot exclude that the apparent increase in rRNA is due to changes in other RNA subtypes. Accordingly, despite the suggested predictive value of altered rsRNAs for fertility (Hua et al., 2019), we did not observe changes in sperm count, fertilization rate, or litter sizes in the Dex-treated sperm and resulting offspring (Figure S6).

**Table 1:** Changes in miRNAs and CircRNAs across time and sample type

Time point after injection	3 hr	7 days	14 days		
**miRNAs**	Cauda	Cauda	Cauda	Caput	Serum
3535	no ch.	no ch.	↑	not det	not det
677-5p	no ch.	no ch.	↑	no ch.	not det
677-3p	no ch.	no ch.	↑		
34c-5p	no ch.	no ch.	↓	no ch.	no ch.
7033-5p	no ch.	no ch.	↑		
703	no ch.	no ch.	↑		
5126	no ch.	no ch.	↑		
5121	no ch.	no ch.	↑		
6240	no ch.	no ch.	↑		
5114	no ch.	no ch.	↑		
1839-3p	no ch.	no ch.	↑		
1949	no ch.	no ch.	↑		
196a-5p	no ch.	no ch.	↑		
3064-5p	no ch.	no ch.	↑		
196b-5p	no ch.	no ch.	↑		
6538	no ch.	no ch.	↑	no ch.	no ch.
18a-5p	no ch.	no ch.	↓		
3963	no ch.	no ch.	↓		
471-5p	no ch.	no ch.	↓		
5099	no ch.	no ch.	↑		
1843b-3p	no ch.	no ch.	↑		
1843a-3p	no ch.	no ch.	↑		
**circRNAs**					
Tasp1			↑	no ch.	
Dennd1b			↑	no ch.	

Table depicts significantly altered small RNAs and circRNAs in cauda sperm at 14 days after dexamethasone injection.

**Table 2:** Changes in tsRNAs across time and sample type

Time point after injection	3 hr		7 days	14 days		
tsRNAs	Cauda	Caput	Cauda	Cauda	Caput	Serum
**Leu-CAA-2**	↑		↑	no ch.	no ch	
*Thr-TGT-1*	↓		↑	no ch.		
*His-GTG-2*	no ch		↓	no ch	no ch	no ch.
*His-GTG-3*	no ch		↑	no ch.		
*Pro-AGG-1*	no ch		↑	no ch.		
*Pro-TGG-2*	no ch		↑	no ch.		
*Pro-TGG-4*	no ch		↑	no ch.		
*Glu-CTC-4*	no ch		↑	no ch.		
*Pro-CGG-1*	no ch		↑	no ch.		
*Gly-GCC-6*	no ch		↑	no ch		
Gly-GCC-2	no ch		no ch	↓	no ch	no ch.
**Arg-CCT-2**	↑	no ch.	↑	↑	↑	↑
Ala-TGC-2	no ch		no ch	↓		
Arg-ACG-3	no ch		no ch	↓		
Glu-CTC-3	no ch		no ch	↓		
Ser-TGA-1	no ch		no ch	↓		
Thr-TGT-2	no ch		no ch	↑		
Ser-TGA-2	no ch		no ch	↑		
Ser-AGA-1	no ch		no ch	↑		
Glu-TTC-3	no ch		no ch	↓		
Arg-CCT-1	no ch		no ch	↑		
Ser-AGA-2	no ch		no ch	↑		
Cys-GCA-3	no ch		no ch	↓		
Pro-AGG-3	no ch		no ch	↑		
Lys-CTT-3	no ch		no ch	↓		
Arg-CCT-3	no ch		no ch	↑		
Lys-CTT-3	no ch		no ch	↓		
Trp-CCA-5	no ch		no ch	↓		
Cys-GCA-2	no ch		no ch	↓		
Ala-TGC-5	no ch		no ch	↓		
Gly-CCC-3	no ch		no ch	↓		
Gly-CCC-4	no ch		no ch	↓		
Ala-TGC-5	no ch		no ch	↓		
Thr-AGT-5	no ch		no ch	↑		
SeC-TCA-1	no ch		no ch	↓		
Thr-AGT-7	no ch		no ch	↑		
Glu-CTC-2	no ch		no ch	↓		
His-GTG-1	no ch		no ch	↑		
Arg-CCT-4	no ch		no ch	↑		
Lys-CTT-1	no ch		no ch	↓		
Lys-CTT-2	no ch		no ch	↓		
Lys-CTT-2	no ch		no ch	↓		
Asp-GTC-4	no ch		no ch	↓		
Gly-GCC-5	no ch		no ch	↓		
Thr-TGT-3	no ch		no ch	↑		
Asn-GTT-2	no ch		no ch	↑		
Asn-GTT-4	no ch		no ch	↑		
Asn-GTT-1	no ch		no ch	↑		
Gly-GCC-4	no ch		no ch	↓		
Gly-GCC-2	no ch		no ch	↓		
Thr-CGT-4	no ch		no ch	↑		
Gly-CCC-5	no ch		no ch	↓		
Ala-TGC-6	no ch		no ch	↓		
Glu-CTC-1	no ch		no ch	↓		

No highlight: Significantly altered in cauda sperm at 14 days after dexamethasone injection. In bold: Persistently altered in cauda sperm at 7 days and 3 hr. Italic: Significant interaction between 7 days and 3 hr.

Some recent publications have suggested that sperm miRNAs and tsRNAs are acquired during epididymal transit from caput to cauda (Sharma et al., 2015, 2018; Conine et al., 2018). Furthermore, it was shown that changes in sperm tsRNAs, induced by chronic nutritional challenge, are acquired by uptake of distinct sets of tsRNAs (Sharma et al., 2015). To examine whether the changes observed 2 weeks after Dex injection were also apparent in caput sperm before epididymal transit, we decided to assess a selection of small RNAs in sperm harvested from caput epididymis using q-PCR. Out of 7 selected small RNAs encompassing both tsRNAs and miRNAs, we found 6 unaltered (Figure 1F) in line with the assumption that epididymal transit is required to allow epididysomal uptake leading to altered RNA cargo in mature sperm. Yet tsArg-CCT-2 was consistently altered in caput sperm (Figure 1F), indicating that either this change is induced at an earlier transcriptional level during spermatogenesis or certain small RNAs are taken up from exosomes in caput epididymis.

To test these two hypotheses, we examined a small set of RNAs in serum-circulating exosomes. Indeed, we detected increased levels of tsArg-CCT-2, while other tsRNAs and miRNAs did not show altered serum exosome payload (Figure 1G). Thus, it appears that tsArg-CCT-2 is taken up by sperm from exosomes in the caput but that other small RNA changes are not necessarily reflected in the payload of circulating exosomes.

**Figure 2:**
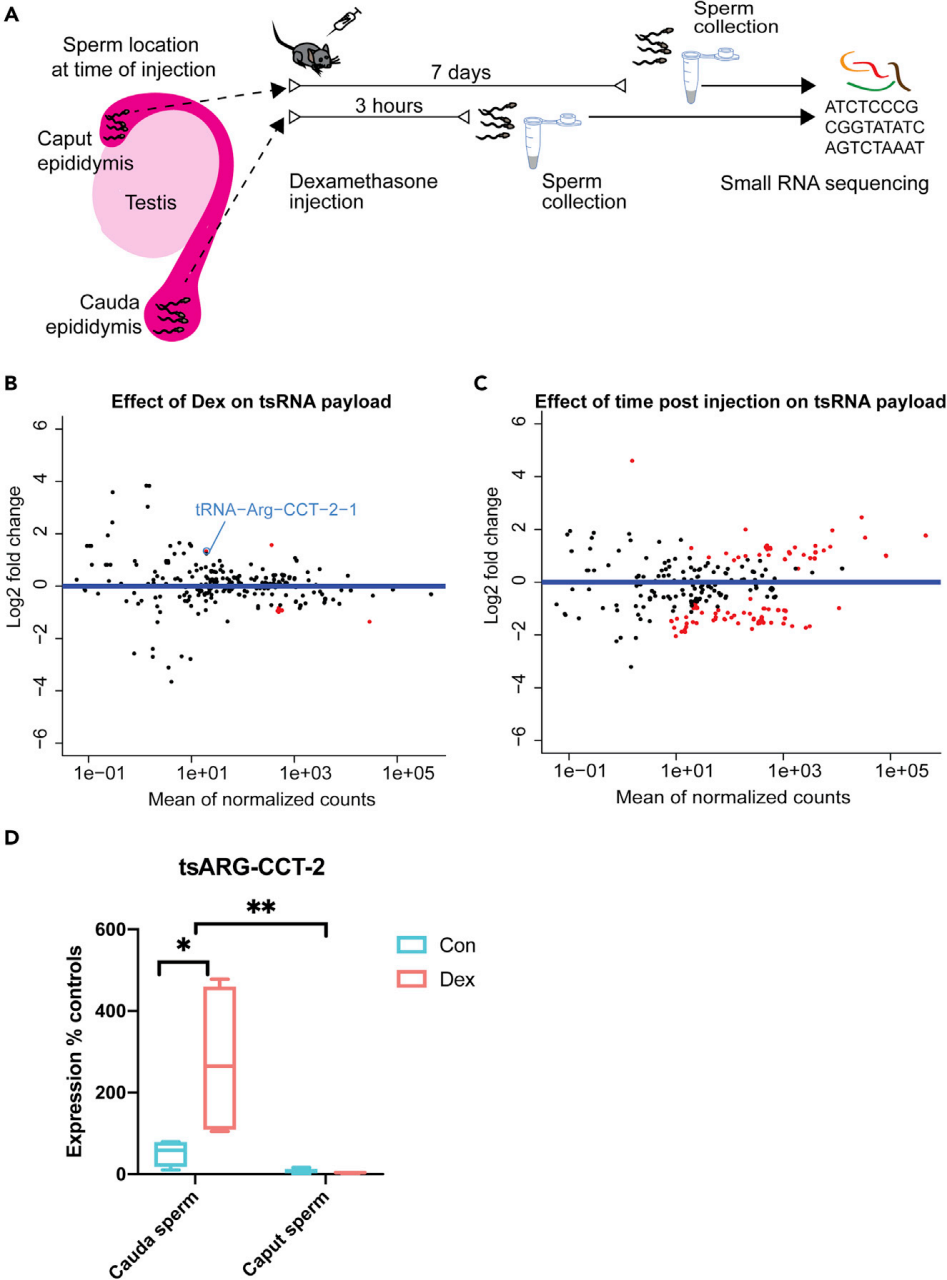
Effect of Dex on sperm cells at different time points post Dex administration (A–C) (A) Experimental design showing the location of sperm at the time of injection and timing of sperm harvest. MA (log-intensity ratios [M-values] versus log-intensity averages [A-values]) plots depicting (B) effect of Dex (log2 fold changes control versus dexamethasone), (C) time after injection (log2 fold changes 7 days versus 3 hr) (7 days Dex n = 4 and controls n = 4, 3 hr Dex n = 3 and controls n = 4). TsRNAs are indicated by sequence identity for display only; each dot represents one small RNA. MA plot depicts log2 fold changes on the y axis and the expression level on the x axis (the higher the expression the further to the right). Statistically significantly changed small RNAs are highlighted in red q < 0.05. (D) Relative expression of ArgCCT-2 as obtained by q-RT-PCR (cauda: Dex n = 4, controls n = 4, caput: Dex n = 4, controls n = 5; interaction F(1,13) = 6.34, p = 0.0257, treatment F(1,13) = 5.97, p = 0.0040, site of collection (F(1,13) = 12.15, p = 0.0296; cauda control versus cauda Dex t(13) = 3.42, p = 0.0274, cauda Dex verus caput Dex t(13) = 4.137, p = 0.007). Whiskers display minimum and maximum. ∗p < 0.05, mixed-effect model group effect of location (REML), ∗∗p < 0.01 multiple comparisons SIDAK corrected.

While the necessity of epididymal transit to acquire changes represents one explanatory framework for the absence of change in all but one selected small RNA in caput sperm, alternative explanations should also be considered. Our results could also indicate that changes observed in mature sperm 14 days after Dex injection represent a highly specific snapshot in time, which relies on the affected sperm cells to be in a specific developmental stage at the time of treatment. Cells entering into more mature stages of sperm differentiation at a later point after Dex administration, such as the cells assessed here sampled from caput, would then no longer display said changes. Therefore, we cannot conclusively establish a reliance on epididymosomal uptake. To further dissect the dependence on epididymosomal uptake during transit from caput to cauda epididymis from a different angle, we assessed the mature sperm small RNA payload at two time points, 3 hr and 7 days after injection (Figure 2A). Cells collected from cauda 7 days after injection have already exited testes and have had time to pass through the entire epididymal tract before collection. In contrast, cells collected 3 hr after injection have most likely not passed through the corpus epididymis and already reside in cauda epididymis at the time of injection where sperm resides up to 5 days (Meistrich, 1975; Dadoune and Alfonsi, 1984). Importantly, spontaneous ejaculation regularly voids cauda epididymis of sperm, even in the absence of a mating partner (Huber and Bronson, 1980), excluding the retention of “old” mature sperm in cauda for prolonged periods of time. The cells collected 7 days after exposure therefore represent a mixture of cells that might have already resided in the cauda and those cells that indeed passed through the corpus epididymis, yet the spontaneous ejaculation ensures that the sample predominantly contains the latter.

The collected samples were confirmed for their purity and again processed separately to represent sperm from one animal per library. The resulting libraries were analyzed jointly as to test for (1) effects of Dex injection independent of sampling time after injection, (2) effects of sampling time after injection independent of Dex treatment, and (3) effects depending on both Dex injection and the sampling time after injection (interaction). We report an average of 64% mappable reads including 46% of multimappers and observe that tsRNAs were significantly affected by sampling time after injection independent of treatment. This demonstrates the fluctuation of tsRNAs over time in response to external signals such as injections, or potentially due to uncontrollable external inputs from the animal husbandry (Figure 2C). Interaction between treatment and time was statistically significant for 27 tsRNA-mapping loci including Gly-GCC-6-1. All affected tsRNAs are upregulated after 7 days. Twenty-six tsRNAs of them are unchanged after 3 hr, and one tsRNA (Thr-TGT1-1) is downregulated after 3 hr (Table S2 sheet 3, q < 0.05). This finding is consistent with the dominating view that tsRNAs are acquired during epididymal transit from caput to cauda epididymis. However, most tsRNAs that showed a significant change in response to treatment after 7 days, but not after 3 hr (interaction between treatment and time post injection, Table S2 sheet 3, q < 0.05), were not persistently altered in the data set of 14 days after injection (Table 1). This indicates that on the one hand changes in sperm RNA are dynamic and many do not persist for prolonged time. On the other hand, this suggests that potentially relevant small RNA changes mostly require either sperm to reside in testis at the time of exposure or rely on a prolonged residency in the exposed organism. Interestingly, we also detected 2 exceptions that show a significant group effect across 3 hr and 7 days. tsRNA-Leu-CAA and tsRNA-Arg-CCT (Figure 2B) were persistently affected 3 hr and 7 days after exposure, which necessarily requires a mode of rapid acquisition of tsRNA changes in cauda epididymis. While the change in tsRNA-Leu-CAA was temporary and did not persist, strikingly tsRNA-Arg-CCT-2 deregulation persisted until 14 days after injection (Figure 1C). To additionally validate the Dex-induced change of tsRNA-Arg-CCT-2 independent of epididymal transit from caput to cauda, we replicated the effect observed in mature sperm sampled from cauda epididymis 3 hr after injection using q-PCR (Figure 2D, Table S2). Additionally, we sampled caput sperm 3 hr after injection and measured tsRNA-Arg-CCT-2 levels. An overall two-way ANOVA revealed a significant interaction between sperm sampling location (caput versus cauda) and treatment (vehicle versus Dex). Post hoc tests confirm a significant increase in tsRNA-Arg-CCT-2 levels in response to Dex in cauda but not in caput sperm and a significant increase in Arg-CCT-2 levels between cauda and caput sperm independent of treatment.

The behavior of miRNAs differed considerably from tsRNAs. As would be expected, if epididymal transit was required for miRNA changes to be implemented, we observe no group effect of treatment on miRNAs (Figure S2A, Table S2) across 3 hr and 7 days after injection. Furthermore, we detected no effect of time after injection on sperm miRNA payload (Figure S2B, Table S2), confirming the absence of an effect of injection on miRNAs per se, nor did we detect an interaction between Dex and time after injection (Figure S2C, Table S2) in miRNAs 7 days and 3 hr after injection, however. Importantly, when inspecting those miRNAs that were significantly altered 14 days after injection, no alterations were apparent 3 hr or 7 days after injection (Figure S2D), indicating that changes in miRNAs occur more slowly or require sperm cells to reside in the testes at the time of injection.

### Effects of acute GR activation on *in vivo* offspring metabolic phenotype

**Figure 3:**
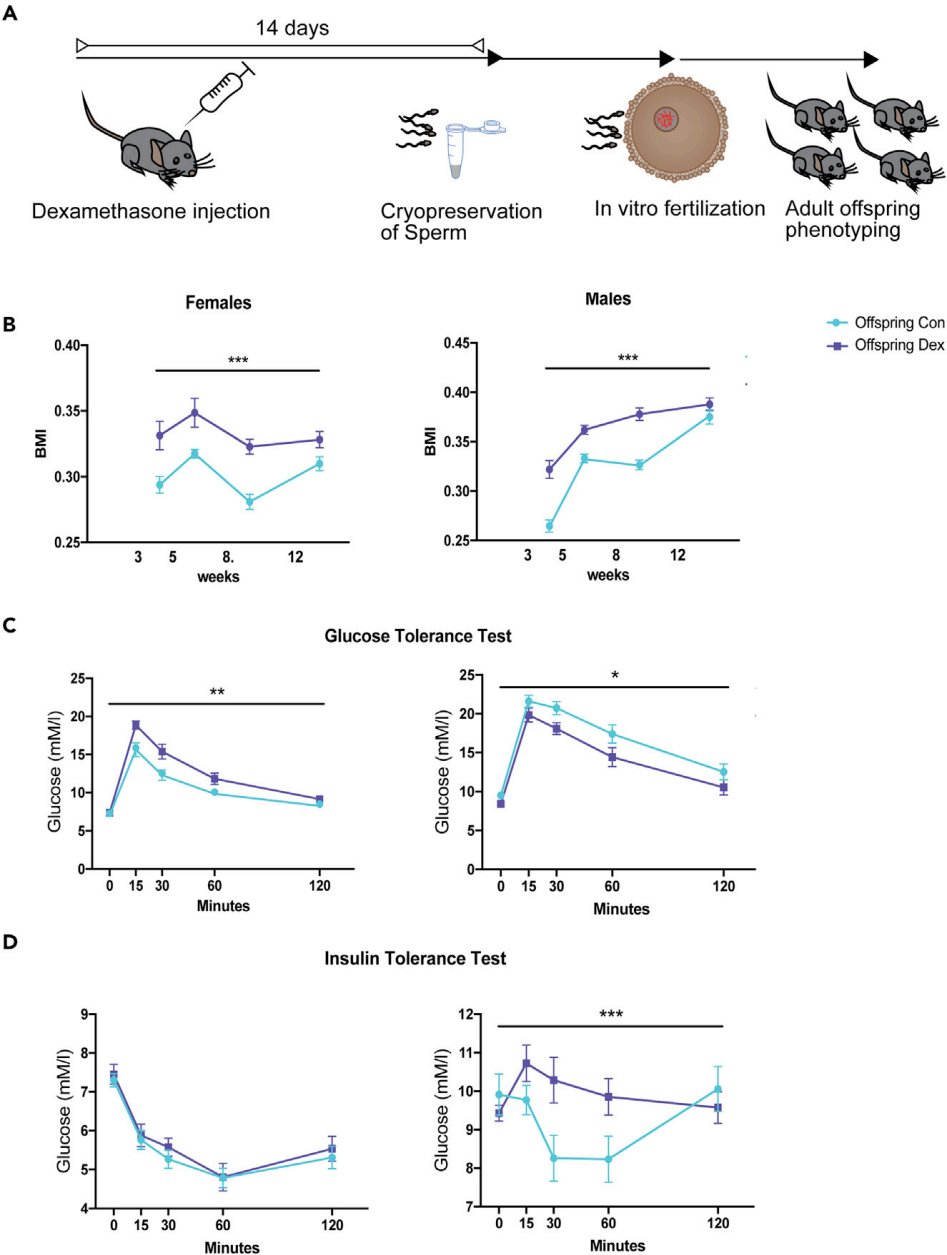
Effect of Dex on metabolic phenotype in the offspring (A) Experimental design depicting timeline between injection, sperm harvest, *in vitro* fertilization, and phenotyping. (B–D) (B) Impact of Dex on male and female adult offspring. (B) Body mass index (males vehicle offspring n = 21, Dex offspring n = 22, females vehicle offspring n = 17, Dex offspring n = 17), (C) glucose tolerance (males vehicle offspring n = 12, Dex offspring n = 12, females vehicle offspring n = 12, Dex offspring n = 12), and (D) insulin tolerance (males vehicle offspring n = 9, Dex offspring n = 8, females vehicle offspring n = 12, Dex offspring n = 12). Error bars display SEM. Detailed statistical results are depicted in Figure S5, and raw data are provided in Table S3). ∗p < 0.05, ∗∗p < 0.01, ∗∗∗p < 0.001, 3-way repeated-measures ANOVA.

Based on the two reports on effects of single foot shock on offspring weight and the impact of a single GR activation on germline small RNA payload, we hypothesized that this acute impact on the receptor is sufficient to elicit intergenerational effects. We thus injected Dex once intraperitoneally, then harvested sperm 14 days after injection, and performed *IVF* using naive oocytes to generate offspring for phenotyping (Figure 3A). Dex treatment did not affect sperm count, fertility rate, or resulting litter sizes (Figure S6).

The weight and size of pups was measured every 2 to 4 weeks starting at weaning (3 weeks of age) until adulthood (12 weeks of age), and the body mass index (BMI) was calculated as a ratio of weight and squared length. Overall ANOVA of the resulting offspring showed a significant effect of treatment ((F1.71) = 76.55, p < 0.0001), time after injection ((F2.087,144.7) = 41.99, p < 0.0001), and sex (F (1, 71) = 76.55, p < 0.0001) on BMI and a significant interaction between time and sex (F (3, 208) = 33.75, p < 0.0001) and time and treatment (F (3, 208) = 5.834, p = 0.0008) (Figures 3B and S3C, Table S3). These results show that while males had generally higher BMI, both male and female offspring of Dex-injected fathers had a higher BMI.

To further explore potential causes and consequences of altered BMI, adult animals were additionally tested for their glucose tolerance after glucose injection. Overall ANOVA of blood glucose levels revealed a significant effect of sex (F (1, 44) = 54.80, p < 0.0001) and time after injection (F (2.593, 114.1) = 196.6, p < 0.0001) and significant interactions between sex and time after injection (F (4, 176) = 6.115, P = 0.0001) and sex and treatment (F (1, 44) = 15.62, P = 0.0003) (Figure S3C). Follow-up repeated-measurements ANOVA separated by sex showed a significant effect of treatment, time, and interaction in females (treatment: F (1, 22) = 12.35, p = 0.0020; time: F (4, 88) = 110.1, p < 0.0001; interaction: F (4, 88) = 2.835, p = 0.0291) and significant effects of treatment and time but no interaction in males (treatment: F (1, 22) = 6.019, p = 0.0225; time: F (4, 88) = 96.36, p < 0.0001; F (4, 88) = 0.5401, p = 0.7067; Figure 3C). These data hence demonstrate a sex-dependent effect of paternal Dex injection on glucose tolerance, with impaired tolerance in females and decreased glucose levels in males in response to glucose challenge.

In addition, blood glucose levels were assessed during the insulin tolerance test. Overall ANOVA showed significant effects of sex (F (1, 37) = 162.6, P < 0.0001) and time (F (3.314, 122.6) = 23.85, P < 0.0001) and revealed a significant interaction between sex and time (F (4, 148) = 12.49, P < 0.0001), time and treatment (F (4, 148) = 5.380, P = 0.0005), and time and treatment and sex (F (4, 148) = 5.392, P = 0.0004) (Figure S3C). Follow-up repeated-measurements ANOVA separated by sex showed a significant effect of time (F (2.982, 65.60) = 44.73, p < 0.0001), yet neither significant effect of treatment (F (1, 22) = 0.3465, p = 0.5621) nor an interaction between time and treatment (F (4, 88) = 0.1373, p = 0.9681) in females (Figure 3D). In males, we observe no effect of treatment (F (1, 15) = 1.467, p = 0.2446) yet detected a significant effect of time after injection (F(2.914, 43.71) = 4.538, p = 0.0079) and a significant interaction between treatment and time after injection (F(4, 60) = 7.003, p = 0.0001, Figure 3D). These results indicate sex- and time-dependent effects of paternal Dex on insulin tolerance. They further show no change in insulin tolerance in female descendants of fathers injected with Dex, yet impaired insulin tolerance in male progeny.

Lastly, we explored a potential reflection of altered BMI in tissue composition by necropsy and weighing the dissected organs and fat pads. Overall ANOVA of necropsy weights revealed a significant effect of sex (F (1, 140) = 28.27, P < 0.0001), tissue (F (4, 140) = 232.7, P < 0.0001), and a significant interaction between sex and tissue (F (4, 140) = 3.379, P = 0.0113) yet no effect of treatment (F (1, 140) = 0.2587, P = 0.6118) or interaction between treatment and sex (F (1, 140) = 0.0004794, P = 0.9826) or treatment and tissue (F (4, 140) = 0.1635, P = 0.9565) on tissue weight (Figures S3A–S3C). This confirms sex dependency, yet no effect of paternal Dex injection on tissue weight in both sexes.

### Effects of acute Dex on offspring early embryonic small RNA

**Figure 4:**
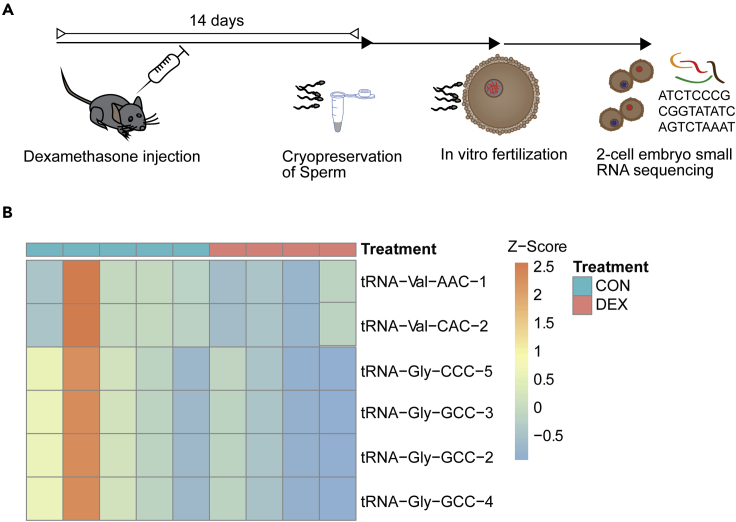
Effect of paternal Dex injection on embryonic offspring small RNA (A) Experimental design depicting timeline between injection, sperm harvest, *in vitro* fertilization, and small RNA sequencing at 2-cell stage. (B) Heatmap showing effect of paternal Dex on small RNA tsRNAs (vehicle embryonic offspring n = 5, Dex embryonic offspring n = 4). TsRNAs are grouped by sequence identity for display only.

The small quantity of paternal RNAs in the zygote relative to the large pool of maternal RNAs poses serious obstacles to their accurate quantification (Chen et al., 2016b). While initial reports on small RNA transmission relied on comparative sequencing or microarray analyses of unfertilized oocytes and fertilized zygotes (Ostermeier et al., 2004), today we are aware that such comparisons can be deceiving, as they rely heavily on both assessment method (e.g., microarray restricted to a selective set versus unbiased genome-wide sequencing) and sequencing depth (Dard-Dascot et al., 2018; Yeri et al., 2018). One such example is inconsistent results regarding miRNAs that are exclusively supplied from the sperm, such as miR-34c, −99a, −214 (Amanai et al., 2006; Liu et al., 2012). Alternative approaches have used indirect measures, e.g., assessing mRNA targets of paternally derived small RNAs (Amanai et al., 2006; Tang et al., 2007; Krawetz et al., 2011; Sharma et al., 2015). We attempted to directly examine the relative difference between the small RNA landscape in early embryos resulting from IVF of naive oocytes with sperm from either Dex- or vehicle-injected males (Figure 4A). We used small RNA sequencing to compare 2-cell embryos derived from Dex-treated or control fathers. We detected an average of 29% mappable reads including 21% multimappers. While we only detected subtle changes in miRNAs of Dex-exposed progeny, we observed downregulation of several tsRNAs from 6 different genomic locations (q < 0.1) (Figure 4B). Strikingly, two of the downregulated tsRNAs (Gly-GCC at several genomic loci and Gly-CCC) were consistently downregulated in sperm 14 days after Dex injection. This could either indicate a reduced delivery of this sperm RNA cargo in Dex-treated males to the oocytes they fertilize or earlier usage and function of respective RNA in Dex leading to a quicker elimination or shorter half-life.

### Effects of acute Dex administration on offspring early embryonic transcriptome

**Figure 5:**
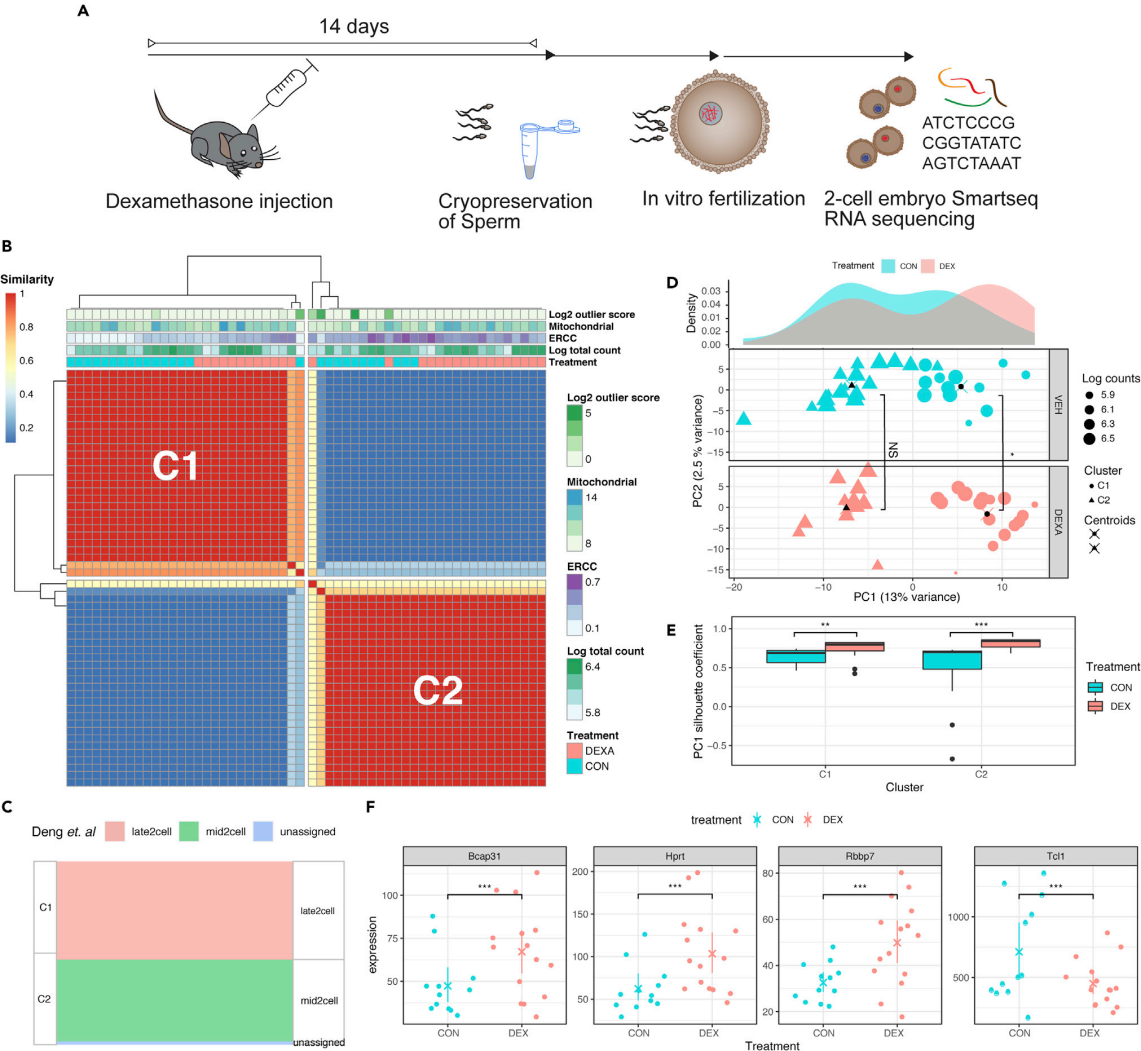
Effect of paternal Dex injection on embryonic offspring long RNA transcriptome (A) Experimental design depicting timeline between injection, sperm harvest, *in vitro* fertilization, and Smartseq2 sequencing at 2-cell stage. (B) Consensus matrix representing the similarity between cells as reported by SC3. Similarity 0 indicates that a given pair of embryos was never assigned to the same cluster, whereas similarity 1 means that a pair of embryos was always assigned to the same cluster. (C) Sankey diagram showing projection of the obtained clusters (C1 and C2) into clusters reported by Deng et al. for single cells obtained from 2-cell embryos. (D) Principal component analysis of two-cell embryos gene expression. The top panel indicates the density of 2-cell embryos along PC1 grouped by condition; control (red) and Dex treatment (blue). The two bottom panels show the distribution of 2-cell embryos across PC1 and PC2 for control (red) and treated (blue) groups. The cluster membership of each embryo is denoted by the point shapes (C1 cycles; C2 triangles), and the centroids of each cluster are indicated with a black symbol overlaid with an x. Wilcox tests were performed to assess differences on PC1 values of C1 and C2 clusters between the treated and control groups. NS denotes nonsignificant change for C2 cluster, while ∗ indicates a significant difference for C1 cluster (p value< 0.05). (E) Silhouette coefficient comparison between treatment and control, statistical significance was assessed with Wilcox test (∗∗ p value < 0.01; ∗∗∗ p value < 0.005) (F) Selection of differentially expressed genes as determined by Monocle within C1 corresponding to late 2-cell embryo stage (∗∗∗ adjusted p value < 0.005). Error bars display 95% nonparametric confidence intervals.

If sperm RNA were directly impacting the zygotic mRNA pool or if they were affecting early embryonic gene expression, then this should be apparent in the 2-cell embryo's transcriptome (Figure 5A). To examine the effect of paternal Dex on early embryonic RNA content, we subjected 2-cell embryos to the Smartseq single-cell sequencing protocol (Figure S4). After performing quality control and filtering the sequenced 2-cell embryo data on criteria such as minimal read count/embryo (Figure S4A), we carried out unsupervised clustering based on their gene expression profiles using SC3 (Kiselev et al., 2017). We identified two distinct clusters (C1 and C2), which were composed by a balanced mixture of treated and control cells (Figure 5B). Since the resolution of single-cell experiments allows characterizing distinctive transcriptomic profiles within early cell division stages, we used scmap (Kiselev et al., 2018), to project each 2-cell embryo gene expression profile onto a reference data set of single cells from 2-cell embryo states previously reported by Deng et al. (Deng et al., 2014) (Figure 5C.) Most of the 2-cell embryos belonging to cluster C1 projected to the late 2-cell stage, whereas embryos from C2 exclusively projected to cells from the mid 2-cell stage. This shows that the two clusters identified through unsupervised clustering correspond to 2-cell embryos in the mid and late 2-cell stage, respectively.

Principal component analysis (PCA) revealed a prominent separation between C1 and C2 along the PC1 axis, suggesting a correlation between PC1 and developmental transitions between mid and late single-cell embryos (Figure S4B.) Interestingly, 2-cell embryo offspring of males injected with Dex exhibited a significant shift of the C1 cluster across PC1 (two-sided Wilcox test p < 0.03), while the C2 clusters did not show significant differences across PC1 between treatment and control groups (Figure 5D.) These results suggest that the effect of paternal Dex treatment on the transcriptome only becomes apparent at the late 2-cell embryo stage. To further explore this hypothesis, we calculated the silhouette coefficient (Rousseeuw, 1987) on PC1, as a measure of distance between C1 and C2 clusters, for the control and treatment group. We observed a significant increase of PC1 silhouette coefficient between treatment and controls for both C1 (one-sided Wilcoxon test p value <0.005) and C2 (one-sided Wilcoxon test p value < 2x10^−5^.) This confirms that Dex treatment affects embryonic gene expression, promoting altered late 2-cell embryo stages, since the divergence from mid 2-cell embryos is significantly bigger in Dex offspring than in control offspring (Figure 5E.)

Accordingly, differential gene expression analysis using Monocle2 (Qiu et al., 2017) focused on late 2-cell embryos (cluster C1) revealed significant gene expression changes between offspring of males injected with Dex and controls across 38 genes, some of which were already apparent to a less significant extent during mid 2-cell embryos (cluster C1; e.g. Tcl1; Table S4) In line with a potentially altered developmental trajectory becoming apparent in cluster 1, the late 2-cell stage includes several affected genes that are involved in early embryonic development. For example, Bcap31 (B-cell-receptor-associated protein 31) is an important element for endoplasmic reticulum (ER) and Golgi apparatus function, and Bcap31 mutations lead to developmental diseases with metabolic disturbances (Cacciagli et al., 2013). This is reminiscent of the metabolic phenotype observed in the adult offspring of Dex-injected fathers. Hypoxanthine-guanine phosphoribosyltransferase (Hprt) is crucial for cell cycle division, and T cell leukemia/lymphoma (Tcl1) regulates cell proliferation (Kang et al., 2013; Miyazaki et al., 2013). Hence, an upregulation of Hprt and a concomitant downregulation of Tcl1 might indicate that cell fate decisions later during development may be affected. Another differentially expressed gene is Rbbp7 (RB-binding protein 7), which is part of many histone deacetylase complexes such as Nurd and PRC2/EED-EZH2 and thus plays an essential role in chromatin-mediated gene regulation (Yu et al., 2018). Interestingly, several forms of PRC mutations in humans lead to different kinds of overgrowth phenotypes (Deevy and Bracken, 2019), an abnormality reminiscent of the increased BMI observed in Dex offspring (Figure 3B.)

### Effects of Dex administration on an interesting candidate for sperm-RNA-mediated inheritance

**Figure 6:**
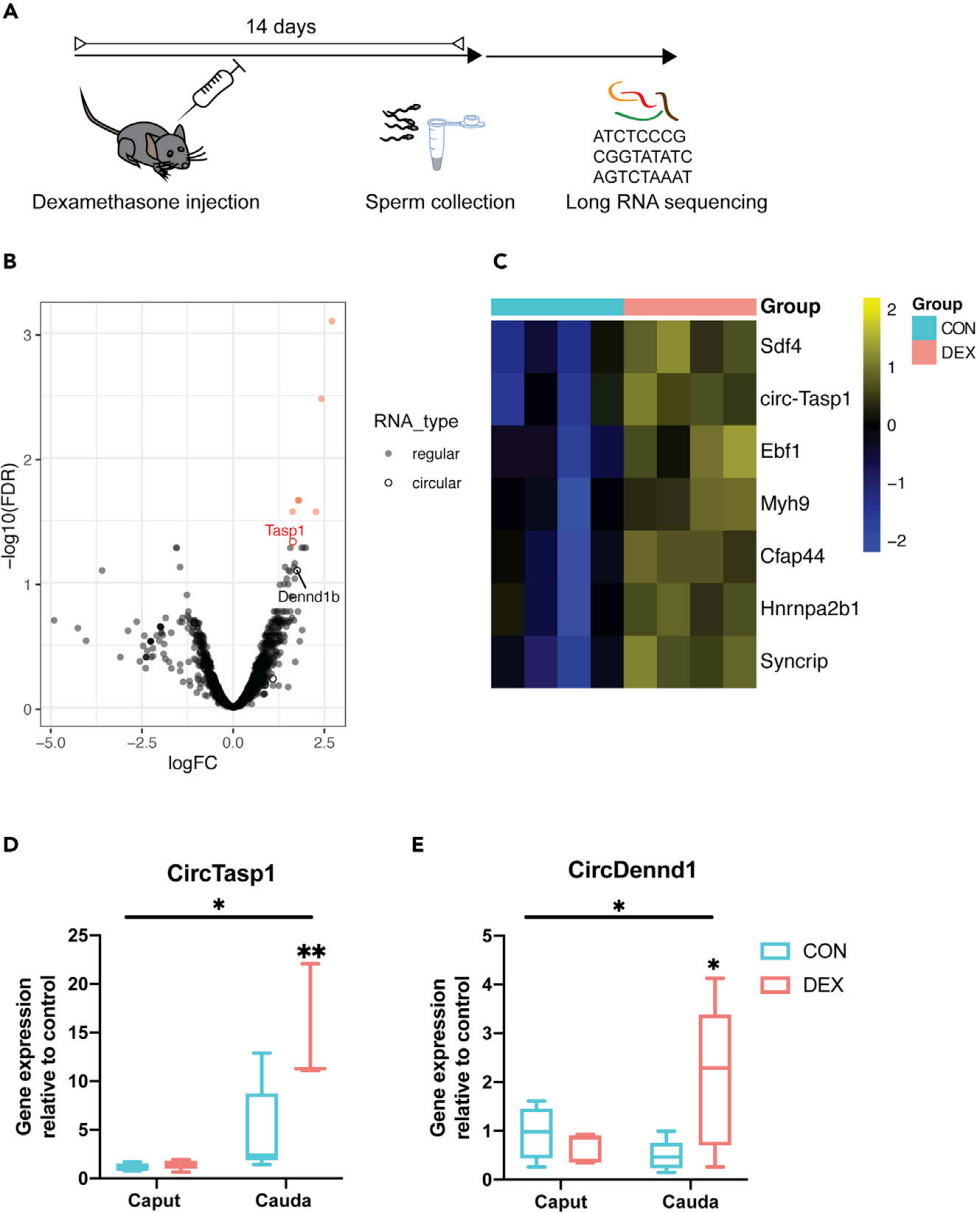
Effects of Dex on long RNA payload of sperm cells residing in testes at the time of administration (A) Experimental design depicting a time window of two weeks between injection of Dex and sperm collection for molecular analysis. (B) Volcano plot depicting fold changes and significance level of long RNA in mature sperm 14 days after injection of Dex (n = 4) versus vehicle (n = 4) as assessed by small RNA sequencing. Statistically significant transcripts are highlighted in red (FDR <0.05). (C) Heatmap showing significantly differentially expressed long RNA transcripts of the same experiment (multiple comparison corrected, q < 0.05). (D) Q-PCR results of CircRNA assays for caput and cauda sperm for Tasp1 (interaction treatment x tissue F(1,5) = 7.53, p < 0.05, caput: control n = 4, DEX n = 5, p > 0.05; cauda control n = 5, DEX n = 3, p < 0.01) (∗p < 0.05 mixed-effect model interaction [REML], ∗∗q < 0.01 multiple comparisons SIDAK corrected) and Dennd1 (interaction treatment x tissue F(1,15) = 6, p < 0.05, caput: control n = 4, DEX n = 5, p > 0.05; cauda control n = 5, DEX n = 5, p < 0.05) (E). Whiskers display minimum and maximum. ∗p < 0.05 mixed-effect model interaction (REML), ∗q < 0.05 multiple comparisons SIDAK corrected.

Despite the observed changes in sperm tsRNAs after acute Dex injection, we did not find an obvious causal connection to the altered 2-cell embryonic transcripts. This prompted us to investigate whether other germline changes might be more crucial for the offspring's *in vivo* alterations in our model. We previously showed that chronic stress exposure also led to changes in sperm-long RNAs that contributed functionally to the transmission of effects to the offspring (Gapp et al., 2018). Yet the fact that sperm RNA is stable through transmission and that the minute amounts of transmitted paternal RNA can elicit major changes in the embryo remains puzzling. Therefore, we evaluated the impact of Dex injection on the highly stable class of circRNAs in male sperm. CircRNAs were previously detected in swine (Gòdia et al., 2020) and human sperm (Chioccarelli et al., 2019) and suggested to have functional implications in epigenetic regulation. They have been attributed a critical role in the male germline after cessation of transcriptional activity (Tang et al., 2020). Using Circexplorer in combination with EdgeR, analysis of sperm-long RNA sequencing of males treated with Dex and controls revealed significant upregulation of two circRNAs (Figure 6A, q < 0.1), and we also observed several significant changes in the sperm-long RNA protein coding transcripts after acute Dex treatment (Figures 6B, 6C, and S1B, Table S5). Both circRNAs are hosted in genes relevant for immune function (Taspase 1: Tasp1 and DENN Domain Containing 1B Dennd1b), yet the host genes did not show differential abundance of the protein coding transcript (Table S5). We then replicated the upregulation of these to CircRNAs by q-PCR in cauda epididymal sperm of a separate set of animals using CircRNA specific primers that span the backsplice junction. At the same time, we also assessed their abundance in caput epididymal sperm to evaluate whether the observed effect was also present in sperm cells under development. We observed a significant interaction between treatment and tissue. Post hoc tests confirm the increased abundance of CircTasp1 and CircDend1 in cauda sperm detected in the sequencing analysis. No change was detectable in caput sperm, arguing against an induction of the change in developing sperm cells (Figures 6D and 6E). Given the absence of transcription, these data suggest that in control conditions, these CircRNAs exert their function during spermiogenesis (for example, by being translated) and that in contrast, in Dex-treated cells, the CircRNA is not consumed to the same extent leading to an apparent upregulation in mature cauda epididymal sperm. CircAtlas (Wu et al., 2020) revealed several potential miRNA sponge targets to be captured by the altered circRNAs. Some of these miRNAs are common sponge targets of both circRNAs such as mir3110-5p, mir706, and mir1955 (Figure S5). Diamine acetyltransferase 1 (Sat1), one of 3110-5p′s high-confidence miRNA-targets, as predicted by TargetScan (Agarwal et al., 2015), is indeed significantly upregulated in the embryos composing cluster 1 (later developmental stage). MiRNA target upregulation is expected if mir3110-5p was downregulated through circRNA-mediated sponging and highlights a potential effective contribution of increased circRNA in sperm to embryonic pathway regulation. This is the first report of a change induced by environmental exposure in this compelling class of RNA in sperm.

## Discussion

By generating offspring using assisted reproductive techniques (IVF), we circumvent potential confounding variables such as transmission via RNA contained in seminal exosomes (Vojtech et al., 2014) and affected maternal care by altered mating behavior (Mashoodh et al., 2012) and prove germline dependence (Bohacek and Mansuy, 2017). Consistent with the significant changes of miRNAs and tsRNAs in the germline 2 weeks after GR activation, previous studies including our own have observed regulation of mouse sperm small RNA in a variety of contexts (Rodgers et al., 2013; Gapp et al., 2014; Grandjean et al., 2015; Sharma et al., 2015; Chen et al., 2016a, 2016b; Wu et al., 2016; Short et al., 2017; Benito et al., 2018; Rompala et al., 2018; Yeshurun and Hannan, 2018).

Especially relevant specifically for our analysis, sperm RNA sequencing after drinking water administration of corticosterone for 4 weeks followed by mating led to strong downregulation of tsRNA-GluCTC and tsRNA CysGCA, two of our top downregulated tsRNAs, indicating that these tsRNAs are responding similarly to acute and chronic insults. At the same time, this chronic manipulation elicits changes of several miRNAs, e.g., 34c and 471 (Short et al., 2016), albeit in the opposite direction of what we find in response to acute Dex treatment. These discrepancies may arise either by the Dex-induced short-term suppression of internal corticosteroids (Barden, 1999) or due to adaptations in response to chronic administration.

While four (Petropoulos et al., 2014; Short et al., 2016; Wu et al., 2016; Cartier et al., 2018) out of five (Bönisch et al., 2016) previous studies did report phenotypic effects after chronic paternal Dex exposure, only two assessed sperm small RNAs to associate the alterations to the sperm RNA payload (Bönisch et al., 2016; Cartier et al., 2018) yielding conflicting outcomes.

These differences might be due to inconsistent life stages (adulthood versus gestational), sperm collection (swim up, somatic lysis, or no purification), and/or dosage of exposure. Depending on the dosage and timing, the complex autoregulation of the GR can lead to GR downregulation after prolonged activation (Gjerstad et al., 2018). Acute exposures have the advantage of avoiding such long-term feedback regulation and hence provide an elegant approach for studying the signaling pathways leading to germline changes.

Mature sperm tsRNAs and miRNAs have been shown to be acquired during epididymal transit (Sharma et al., 2015, 2018), and miRNAs are necessary for early embryonic development under certain circumstances (Liu et al., 2012; Conine et al., 2018) (Zhou et al., 2019) (Wang et al., 2020). Furthermore, a recent publication suggests that chronic-stress-induced sperm miRNAs are taken up primarily from epididymosomes originating from the caput epididymis or the proximal epididymal tract (Chan et al., 2020). Chronic nutritional manipulation with effects on offspring also suggests the necessity of epididymal transit to acquire tsRNA changes in sperm (Sharma et al., 2015). Harvesting mature sperm 3 hr after exposure yields a population enriched for cells that had been exposed while already in the cauda epididymis, where spermatids reside for roughly 5 days (Meistrich, 1975). These cells have not traveled through the epididymis, nor have they had a chance to potentially take up small RNA from caput-derived epididymosomes after Dex administration. As expected, we detect no changes in miRNAs in these samples. We do however detect changes in tsRNAs 3 hr after Dex treatment, some of which even persist 14 days after injection. These results show rapid acquisition of changes *in vivo* and corroborate previous *in vitro* findings that show that incubation with epididymosomes can alter sperm RNA payload (Sharma et al., 2015). Our acute intervention assesses effects on germline payload already after a short interval, whereas chronic interventions – based on their experimental design – do not assess changes in mature sperm soon after the first intervention. Studies aiming at the elucidation of the origin of sperm RNA changes might benefit from acute interventions to circumvent confounders such as dynamic exosomal RNA supply as a result of cumulative interventive strain on animals.

An additional option for sperm RNA alterations in transcriptionally inert sperms was suggested in a recent study that found mitochondrial tRNA cleavage in the T-loop in response to a one-week high-sugar diet in humans (Nätt et al., 2019). In line with this observation, Dex injection could trigger oxidative stress (Bera et al., 2010) which provokes such cleavage to increase tsRNA levels (Thompson et al., 2008). A role for oxidative stress in sperm RNA dynamics is further supported by a recent study in boar sperm that found seasonal differences in sperm small and long RNA associated with changed abundance of transcripts mapping to oxidative-stress-, DNA-damage-, and autophagy-related genes (Gòdia et al., 2019). However, such potentially oxidative-stress-mediated mechanism does not explain a rapid decrease of tsRNAs 3 hr after Dex injection.

Importantly, we show alterations in sperm tsRNAs that persist in the oocyte concomitant with changes in early embryonic gene expression and a metabolic phenotype in adulthood. tsRNAs and tRNA-Gly-derived fragments in particular are known to induce chromatin-structure-mediated gene regulation and to regulate cell differentiation in various contexts (Li et al., 2016; Guzzi and Bellodi, 2020). Hence, we propose that the transmitted reduction in key tsRNAs such as Gly-GCC and Gly-CCC explains in part the observed perturbations during late two-cell embryo developmental stage. This might reflect an accelerated developmental transcriptional program in the preimplantation embryo of Dex-injected males, ultimately resulting in aberrant BMI and glucose metabolism later in life.

Additionally, we have discovered alterations in circRNA abundance in mature sperm that might also impact the developmental program in the early embryo. circRNAs have the potential to be translated into proteins via backsplicing (Legnini et al., 2017). Accordingly, they are crucial contributors to spermiogenesis after transcriptional cessation because they provide a stable alternative to linear mRNA templates for protein translation (Tang et al., 2020). Transmitted sperm circRNAs could likewise contribute to translation after fertilization, yet the unconventional lattice state of ribosomes preventing normal rates of translation after transcription (Israel et al., 2019) accompanied by a rapid increase in proteins of the ubiquitine/proteasome pathway( Wang et al., 2010) make this unlikely. Nevertheless, a study on human sperm detected abundant levels of circRNA with predicted regulatory function of early developmental genes in sperm heads, suggesting transmission and function after fertilization (Chioccarelli et al., 2019). By sponging miRNAs that regulate early embryonic transcripts, circRNA could amplify minute signals from paternal environment, such as might be the case for the gene transcript Sat1, which displays increased expression in Dex offspring in 2-cell embryos from cluster 1.

Besides altered RNA identity, nucleic acid modifications especially of RNA but also DNA methylation and chromatin accessibility might contribute further to the effects of Dex injections on offspring metabolism. While detection of changes in each player should be subject of further investigation and might reveal a glimpse of their potential implication, proof of the individual relative causal contribution is extremely challenging because they likely require tight interaction to unravel their orchestrated effects.

Finally, it might be useful to consider testing the translatability of our findings to humans. Here we investigated the effects of a single Dex administration soon after the injection in mice, mimicking a single GR activation such as elicited by treatment of an acute asthma exacerbation (Cross et al., 2011). The recent report that Dex can reduce the number of deaths associated with the COVID-19 pandemic (Biggest COVID-19 trial tests repurposed drugs first, 2020) further prompts the re-evaluation of the impact of prolonged Dex treatment on offspring phenotype. From a clinical perspective, additional consideration is warranted for consequences on offspring health when extended time has elapsed between treatment and time of conception. Such designs may pave the way for the extrapolation of our findings.

We conclude that acute Dex treatment can induce germline epimodifications in the form of small and long noncoding RNA, which likely are relevant in the transmission of the effects of single traumatic events on offspring well-being. Our data suggest that sperm small RNAs are not solely regulated via epididymosomal uptake during transition from caput to cauda epididymis. This expands the interpretation from chronic dietary and stress exposures (Sharma et al., 2015; Chan et al., 2020), where uptake of tsRNAs and miRNAs via epididymosomes has been suggested to lead to differential sperm payload, yet required sperm to transit from caput to cauda to bring about the changes. A persistent detection of significant fold changes of the exact same sperm small RNA in the embryo suggests functional implication in the information transfer from father to offspring. Together with potentially transmitted miRNA sponges in the form of circRNAs, this likely contributes to a slight developmental acceleration of gene expression programs in the early embryo and ultimately manifests in a metabolic phenotype. Future studies may aim at testing the causal contribution of specific sperm RNAs to the transmission of effects of acute impacts. Certainly, continuous methodological refinement will help dissect the relative implication and the interplay of the distinct germline modifications such as DNA methylation, histone PTMs, and chromatin architecture in this highly complex process.

### Limitations of the study

This study identifies a highly dynamic response of sperm RNA cargo in response to a single Dex administration and presents altered CircRNAs in mature sperm in response to an environmental insult with consequences on the progeny's metabolism. While our study implies a functional role of altered sperm RNAs in the transmission of a Dex-induced phenotype to the offspring, a causal proof will require RNA injections into fertilized naive oocytes. Second, although we identified one tsRNA to be persistently altered in serum exosomes as well as in caput and cauda sperm and at different times after Dex injection, the identification of the tissue of origin of the altered tsRNAs would require technically highly challenging metabolic labeling experiments.

## STAR★Methods

### Key resources table

**Table:** 

REAGENT or RESOURCE	SOURCE	IDENTIFIER
**Chemicals, peptides, and recombinant proteins**		

Dexamethasone	Sigma	Cat#D4902-25MG
DMSO	VWR	Cat#472301-100ML
Hyperova	Cosmo Bio	Cat#KYD-010-EX-X5
KSOM	Millipore	Cat#MR-121-D
Trizol	Thermo Scientific	Cat#15596026
Directzol	Zymo	Cat#R2080
M-MLV	Promega	Cat#M1701
Recombinant RNAsin Ribonuclease inhibitor	Promega	Cat#N2111
Insulin	Novo Nordisk	Cat#Actrapid

**Critical commercial assays**		

exoRNeasy Serum/Plasma Midi Kit	Qiagen	Cat#77044
Truseq small RNA library kit	Ilumina	Cat#RS-200-0012
Truseq Total RNA library kit	Ilumina	Cat#RS-122-2301
Nextflex small RNA library kit	Perkin Elmer	Cat#NOVA-5132-05
Nextera XT DNA Library Preparation Kit	Ilumina	Cat#FC-131-1024
miRCURY LNA RT Kit	Qiagen	Cat#339340
miRCURY® LNA® miRNA SYBR® Green PCR	Qiagen	Cat#339345

**Deposited data**		

Sperm small and long RNA sequencing data	Gene Omnibus	GSE162112
2-cell embryo RNA sequencing data	ENA	ERP105660

**Experimental models: Organisms/strains**		

C57Bl/6Jrj mice	Janvier lab	
C57Bl/6 CBLT mice	Sanger Institute	

**Oligonucleotides**		

mirCURY LNA miRNA PCR Assay ArgCCT-2 5`GCCCCAGUGGCCUAAUGGAUAAGGCACUGGCC3`	Qiagen	Cat#339317
mirCURY LNA miRNA PCR Assay Gly-GCC-2 5`GCAUUGGUGGUUCAGUGGUAGAAUUCUCGCCU3`	Qiagen	Cat#339317
mirCURY LNA miRNA PCR Assay His-GTG-3 5`GCCGUGAUCGUAUAGUGGUUAGUACUCUGCGU3`	Qiagen	Cat#339317
mirCURY LNA miRNA PCR Assay Leu-CAA-2 5`GUCAGGAUGGCCGAGUGGUCUAAGGCGCCAGA3`	Qiagen	Cat#339317
mirCURY LNA miRNA PCR Assay RnU6	Qiagen	Cat#339317
mmu-miR-677-p5 mirCURY LNA miRNA PCR Assay	Qiagen	Cat#339306
mmu-miR-3535 mirCURY LNA miRNA PCR Assay	Qiagen	Cat#339306
mmu-miR-6538 mirCURY LNA miRNA PCR Assay	Qiagen	Cat#339306
CircTasp1 FW `CTT AGG AGA GAT TGA ATG TGA TGC C` RW `AAA GGG AGT CAA CCA CTC AG`	Microsynth	Cat#4059186 & 4059187
CircDennd1 FW `AGCTTTCCCAGTTTATTGATGGT` RW `GAAGCCACCCGAAGTGATCT`	Microsynth	Cat#4059182 & 4059183

**Software and algorithms**		

Code	Github	https://github.com/ETHZ-INS/Sperm-RNA-Dex).
GraphPad Prism	GraphPad Prism version 8	www.graphpad.com
featureCounts	Liao et al. (2014)	http://subread.sourceforge.net/
DESeq2	Love et al. (2014)	https://bioconductor.org/packages/release/bioc/html/DESeq2.html
edgeR	Anders et al. (2013)	https://bioconductor.org/packages/release/bioc/html/edgeR.html
STAR	Dobin et al. (2013)	https://github.com/alexdobin/STAR
CIRCexplorer2	Zhang et al. (2016)	https://circexplorer2.readthedocs.io
Scater	McCarthy et al. (2017)	https://bioconductor.org/packages/release/bioc/html/scater.html
Monocle	Qiu et al. (2017)	http://cole-trapnell-lab.github.io/monocle-release/
Trimmomatic	Bolger et al. (2014)	www.usadellab.org
Cutadapt	Martin (2011)	https://cutadapt.readthedocs.io

### Resource availability

#### Lead contact

Further information and requests for resources and reagents should be directed to and will be fulfilled upon reasonable request by the lead contact, Katharina Gapp (katharina.gapp@hest.ethz.ch).

#### Materials availability

The study did not generate new reagents.

#### Data and code availability

- The raw datasets supporting the conclusions of this article are included within the article (supplementary tables).
- Sequencing data have been deposited at Gene omnibus and ENA and are publicly available as of the date of publication. All sequencing data were deposited to Gene Omnibus and ENA.
- All codes have been deposited and are publicly available on GitHub.

Accession numbers are listed in the key resources table.

### Experimental model and subject details

#### Animals

C57Bl/6 mice were obtained from the Sanger Research support facility in-house breeding colony. They were housed in a temperature- and humidity-controlled facility in individually ventilated cages under a nonreversed light-dark cycle (Sanger Research Support Facility) or a reversed light-dark cycle (ETH EPIC). Standard chow (LabDiet(r) 5021-3 supplied by IPS) and water were provided *ad libitum after weaning* unless stated otherwise (e.g., oocyte donors). Breeding colony was provided SAFE R03-10 breeding diet, supplied by SAFE diets. Experimental procedures were performed during the animals’ inactive cycle at Sanger. Age- and weight-matched (margin of one week) males were used in each experimental group receiving Dex injections. Animals used for Dex injection followed by sperm sequencing were all sexually mature (14 and 7 days or 3 hours after treatment were 13, 11, and 9 weeks of age, respectively) at the time of sperm collection.

C57Bl/6 males used for sperm sequencing 14 days after Dex injection and q-PCR experiments/validation 3 hours after Dex were obtained from the ETH`s EPIC in house breeding colony in Zürich and were 14-18 weeks old. These mice were fed chow #3734 by Kliba/Granovit.

IVF oocyte donor females and embryo recipients were fed SAFE R03-10 breeding diet, supplied by SAFE diets until 10 days after embryo transfer. Until this time, embryo recipients were housed in pairs after which they were split into single housing. IVF offspring was weaned at PND21 and assigned to cages avoiding littermate cohousing. Offspring phenotyping was carried out between 3.5 to 4 months and necropsy at 4.5 months of age in balanced (offspring controls, offspring treatment) and age-matched groups (all animals had an age spread of 3 days). Animals were housed in groups of 4-5 mice/cage in the Sager Institute barrier research support facility (all animals apart from animals for q-RT-PCR experiments) and ETHZ`s EPIC facility (animals for q-RT-PCR).

All experiments were approved by the UK home office (project license P176396F2) and Cantonal commission for animal experimentation Zürich (project license ZH222/19).

### Method details

#### Dex treatment and sample collection

Age-matched males with an age spread of 1 week were randomly assigned to control and treatment groups. Males were injected with either 2mg/kg of Dex in 10% DMSO 0.9% saline or vehicle (10%D MSO in 0.9% saline). Males used for sperm collection did not undergo any metabolic testing. They were sacrificed 2 weeks, 7 days, and 3 hours after Dex or vehicle treatment. Cauda epididymis and vas deference were dissected and placed in M2 medium. After allowing sperm to diffuse into M2 medium, cells were pelleted by short centrifugation and washed with PBS. For sperm RNA sequencing and q-PCR, mature sperm cells were separated from potential somatic contamination by somatic lysis, followed by 2 washes with PBS (Brykczynska et al., 2010). Sperm counts and fertilization rate appeared unaffected after Dex injection (Figures S6A and S6B).

#### *In vitro* fertilization and embryo culture

Twelve randomly selected, C57BL/6 females were superovulated at 26-31 days of age with Card Hyperova (Cosmo Bio, KYD-010-EX-X5), followed by 7.5 IU human chorionic gonadotrophin (HCG) 48 hours later.

Cumulus-oocyte complexes (COCs) were released from the ampulla of the oviduct 16-17 hours after HCG administration and preincubated in high-calcium HTF with glutathione medium for 30-60 minutes (in CO2 incubator at 37 deg C, 5% CO2 in air) before insemination. Frozen sperm used for insemination was pooled from 2 males that had been injected with Dex or vehicle 14 days prior to cryopreservation. Thawed sperm was preincubated for 30 minutes in TYH (with Methyl-b-cyclodextrin, Sigma C4555) medium at 37 deg C, 5% CO2 in air, before being added to the COC complexes for fertilization. Four hours after insemination, the presumptive zygotes were washed through several drops of KSOM (Millipore, MR-121-D) and incubated overnight in KSOM.

For *in vivo* offspring, 14-20 2-cell embryos from overnight culture in 6 individual IVF dishes/group were implanted into 0.5 dpc pseudo-pregnant F1 females (6 females/group). Each dish contained oocytes from one female with the exception of 2 dishes (out of 6) in the Dex group that contained oocytes of the same female, since one female failed to superovulate. For molecular (single) embryo gene expression analysis at the two-cell stage, 2-cell embryos from overnight culture were frozen and cultured in preincubated KSOM after thawing briefly until/during plating into 96-well plates. The females used to generate these embryos were superovulated with PMSG. The IVF protocol is based on EMMA Harwell’s protocol (adapted from Takeo and Nakagata, 2011) (Nakagata, 2011)), and the sperm freeze protocol is based on the procedure followed by Ostermeier G.C. et al. (2008) (Ostermeier et al., 2008). Resulting litter sizes did not differ between vehicle- and Dex-injected offspring (Figure S6C).

#### Sperm and embryo RNA extraction

Total RNA was prepared from adult mouse sperm using Trizol (Thermo Scientific 15596026) and Directzol (Zymo R2080). Total RNA was prepared from zygotes using the Trizol LS protocol. Quantity and purity of RNA were determined using Agilent 2100 Bioanalyser (Agilent Technologies) and Qubit fluorometer (Life Technologies). Absence of prominent ribosomal peaks indicated absence of somatic cell contamination.

#### Serum exosome RNA extraction

Trunk blood was collected from animals after cervical dislocation and stored at room temperature for 30 minutes to allow coagulation. Serum was subsequently separated by 2 centrifugation steps first for 10 min followed by 15 minutes at 3000. g. One hundred microliters of serum was used as input for exosomal isolation following the manufacturer’s instructions (exoRNeasy Qiagen). Two microliters of 25-ul RNA eluate was used as input for cDNA conversion with the miRCURY® LNA® cDNA conversion kit.

#### Sperm RNA sequencing (RNAseq)

Sequencing was performed using an Illumina Genome Analyzer HiSeq 2500 (Illumina) in Rapid run mode for long 100-bp and small 50-bp RNA sequencing runs.

Libraries for long RNA sequencing were prepared using the TruSeq Stranded Total RNA kit according to the manufacturer's instructions with indices diluted at 1:3. Two hundred nanograms of total sperm RNA was subjected to removal of rRNA using Ribozero gold kit. Approximately 100 ng of sperm RNA and total RNA of several 2-cell zygotes was subjected to TruSeq or Nextflex (sperm 14 days after injection) small RNA library preparation following the manufacturer’s recommendations with the following modifications: adapters were diluted 1:4, and PCR cycles were augmented to 18 and 22 (Nextflex) PCR cycles, respectively. When library preparation of samples was split across days, groups were balanced to circumvent batch effects.

#### Single-embryo seq

Two-cell embryos were generated using the same conditions as indicated for *in vivo* offspring yet followed by embryo cryopreservation until processing for library preparation. They were thawed, and those that appeared intact (34 controls and 37 Dex) were pipetted into wells of 2 96-well culture plates containing lysis buffer and stored at -80°C before processing according to the Smartseq2 protocol and manufacturer’s recommendations (Nextera). Libraries contained a 1:19 Million dilution of External RNA Controls Consortium (ERCC) spike-ins (4456740 Ambion) and were amplified for 18 PCR cycles. Sequencing was performed on a HiSeq V4 under paired end 75bp mode.

#### Insulin and glucose tolerance test

Animals were fasted 4 hours to establish a shared baseline glucose level. They received a single injection of insulin (insulin: 1 mU/g body weight) (Actrapid Novo Nordisk), glucose (2 mg/g body weight), or vehicle (saline) intraperitoneally. Blood samples were taken from the lateral tail vein in adult animals to assess blood glucose level using an Accuckeck aviva device.

#### Body mass index

Animal lengths were measured using a standard ruler and weighed for assessing body weight. The BMI was calculated using the following formula: weight (g)/(length (cm)ˆ2).

#### Necropsy

Organs were dissected after sacrifice and weighed immediately on a scale using “g” as a unit with an accuracy of 2 decimals (accurate down to 10 mg).

#### Small RNA q-RT-PCR

Five nanograms per sample RNA isolated from sperm was reverse transcribed (RT) using the miCURY LNA RT kit (Qiagen #339340). Quantitative RT-PCR (qRT-PCR) was performed using SYBR green based detection in a Biorad thermal cycler with MiRCURY LNA-based small RNA probes designed against tRNA ArgCCT-2, Gly-GCC-2, His-GTG-2, Leu-CAA-2, with a polyA tail directed reverse miRCURY primer (Qiagen # 339317). RnU6 was used as an internal control in sperm samples and mir-103a-3p in serum samples (Qiagen # 339306).

#### circRNA q-RT-PCR

One hundred nanograms per sample RNA isolated from sperm was converted into cDNA using random hexamers. Primers were designed to span the exon splice junctions. Primer sequences for CircTasp1 and circDennd1b *are depicted in the resource table. Tubulin1 was used as endogenous control in the Sybr-Green-based quantification.*

### Quantification and statistical analysis

#### Bioinformatic analysis

##### Bulk RNA sequencing

Each sequencing library represented sperm harvested from a single male. Sequencing quality was assessed with FastQC (Andrews, 2010) and MultiQC (Ewels et al., 2016). Adapters were removed from the 3’ ends with cutadapt (Martin, 2011) (version 1.14), and the resulting sequences with 14 nucleotides of length or less were discarded. All other reads were aligned end to end (no soft clipping) to the ENSEMBL *Mus musculus* genome (release 75) (Flicek et al., 2014) with STAR (Dobin et al., 2013). No mismatches were allowed. Featurecounts was used to match the alignments against the miRbase (Kozomara and Griffiths-Jones, 2011) annotation (version 21) and obtain a matrix of miRNA counts. We applied fractional counts whenever alignment occurred at multiple genomic locations. Differential expression was analyzed using DESeq2 (Love et al., 2014). Quantification of tRNA fragments was performed as above, but all CCA-3ʹ trinucleotides were trimmed after adapter removal, sequences with 15 nucleotides or less were subsequently discarded and GtRNAdb (Chan and Lowe, 2016), and annotation (GRCm38/mm10) was used to obtain the count matrix. Quantification of rRNA fragments was performed using SPORTS (Shi et al., 2018) on the precompiled database included in the tool.

For the data set collected 14 days after Dex injection, library preparation included the insertion of 2 random tetranucleotides between read and adapters. By including only unique sequences in the analysis, we removed duplicates due to PCR amplification.

Long RNAseq libraries were preprocessed with trimmomatic (Bolger et al., 2014) to remove adapters. Reads were aligned to the genome using STAR (Dobin et al., 2013) and quantified using featurecounts (Liao et al., 2014). Circular RNAs were quantified using Circexplorer2 (Zhang et al., 2016) based on junction reads as detected by STAR. Differential expression analysis was performed on the combined set of counts for circular and noncircular RNAs using edgeR (Anders et al., 2013). Robust estimation of dispersion was used to avoid spurious significance due to outliers.

##### 2-cell single-embryo sequencing analysis

Reads from 2-cell embryos were mapped to the mouse reference genome (mm10) and ERCC spike-ins using STAR (Dobin et al., 2013). Resultant alignments were processed to quantify the expression of annotated genes by GENCODE (vM11) and ERCC spike-ins using featureCounts (Liao et al., 2014). To filter low-quality sequenced embryos, we only considered those which had a total read count of at least 0.5 million reads with less than 15% and 10% their read counts mapping to mitochondrial genes and ERCC spike-ins, respectively. After these filters were applied, a total of 56 embryos (29 controls and 27 treated) remained. We clustered their gene expression profiles using SC3 (Kiselev et al., 2017) obtaining two main clusters (C1 and C2). Using scmap (Kiselev et al., 2018), we projected the gene expression profiles for the two-cell embryos onto an index containing expression profiles from zygotic, early/mid/late 2-cell embryos and 4-cell embryo cells reported by Deng et al. (Deng et al., 2014). We performed PCA using scater (McCarthy et al., 2017) (runPCA function), and we calculated the PC1 silhouette coefficient using in-house R scripts. To perform differential gene expression analyses, we normalized the read counts of each embryo as FPKM and we used Census (Qiu et al., 2017) algorithm to convert these values into relative transcripts counts. We computed the obtained ‘Census counts’ using Monocle (v 2.99.2), assuming a negative binomial distribution and a lower detection limit of 0.5. We performed differential gene expression analyses between the total treated and control embryos and also between the treated and control embryos inside of C1 and C2 clusters.

#### Remaining statistical analyses

Sample size for *in vivo* offspring phenotyping was estimated based on previous work on similar models (Hoyer et al., 2013; Bohacek et al., 2016). Three-way repeated-measures ANOVA was used to assess statistical significance for BMI, GTT, and ITT measurements. Necropsy data were analyzed using 3-way ANOVA followed by multiple t-tests and corrected for multiple comparisons using the Benjamini-Hochberg method. Normality was assessed with the Kolmogorov–Smirnov test and met in all necropsy data. Homogeneity of variances was assessed and met in all necropsy data unless gonadal WAT. These t-tests did not assume homogeneity of variances (applied Welchs correction). Q-RT-PCR results comparing caput and cauda sperm RNA were analyzed by fitting a mixed model followed by post hoc tests to compare individual groups applying the Sidak correction for multiple comparisons. Other q-PCRs were analyzed using multiple t-tests corrected for multiple comparisons applying the two-stage step-up method by Benjamini, Krieger, and Yekutieli. All reported replicates were biological replicates. Significance was set at p < 0.05 and where applicable q < 0.05 for all tests. All statistics of behavioral, metabolic tests and q-RT-PCR were computed with Prism. Outliers were removed from q-PCR results using Prismˆs inbuild ROUT method and are depicted in the supplementary tables containing raw data with a star.

## Supplemental information

Document S1. Figures S1–S6

Table S1. Data and analysis of sperm small RNA 14 days post Dex, related to Figure 1 Sheet 1: List of normalized miRNA read counts of sperm harvested 14 days post Dex and vehicle injection. Sheet 2: Deseq2 results of a comparison between miRNA from sperm harvested 14 days post Dex and vehicle injection. Sheet 3: List of normalized tsRNA read counts of sperm harvested 14 days post Dex and vehicle injection. Sheet 4: Deseq2 results of a comparison between tsRNAs from sperm harvested 14 days post Dex and vehicle injection. Sheet 5: List of normalized read counts for rRNA from sperm harvested 14 days post Dex and vehicle injection. Sheet 6: Deseq2 results for rRNA quantification from sperm harvested 14 days post Dex and vehicle injection. Sheet 8: Raw values of qRT-PCR analysis for serum. Sheet 9: Raw values of qRT-PCR analysis of caput sperm.

Table S2. Data and analysis of sperm small RNA 3 hr and 7 days post Dex, related to Figure 2 Sheet 1: Deseq2 results of the comparison of tsRNAs in sperm harvested 3 hr and 7 days post Dex with tsRNAs in sperm harvested 3 hr and 7 days post vehicle injection. Sheet 2: Deseq2 results of the comparison between tsRNAs from sperm harvested post Dex and vehicle injection at 3 hr with tsRNAs in sperm harvested 7 days post Dex and vehicle injection. Sheet 3: Deseq2 results of the interaction between treatment and time for tsRNAs from sperm harvested 3 hr and 7 days post Dex and vehicle injection. Sheet 4: Deseq2 results of the comparison of miRNAs in sperm harvested 3 hr and 7 days post Dex with miRNAs in sperm harvested 3 hr and 7 days post vehicle injection. Sheet 5: Deseq2 results of the comparison between miRNAs from sperm harvested post Dex and vehicle injection at 3 hr with miRNAs in sperm harvested 7 days post Dex and vehicle injection. Sheet 6: Deseq2 results of the interaction between treatment and time for miRNAs from sperm harvested 3 hr and 7 days post Dex and vehicle injection. Sheet 7: Raw values of qRT-PCR analysis for tsRNA-Arg-CCT-2 in caput and cauda sperm sampled 3 hr post Dex and vehicle injection.

Table S3. Raw data for Dex offspring phenotyping, related to Figure 3 Raw data for BMI (sheet 1), GTT (sheet 2), ITT (sheet 3) and necropsy weights (sheet 4) of adult offspring animals resulting from IVF of wildtype oocytes and sperm harvested 14 days post Dex and vehicle injection.

Table S4. Single embryo RNA sequencing analysis, related to Figure 5 Sheet 1: Monocle output list of significantly differentially regulated genes between 2-cell embryos resulting from IVF of wildtype oocytes and sperm harvested 14 days post Dex and vehicle injection.

Table S5. Data and analysis of sperm long RNA 14 days post Dex, related to Figure 6 Sheet 1: List of normalized long RNA seq counts of sperm harvested 14 days post Dex and vehicle injection. Sheet 2: EdgeR results of a comparison between long RNA reads from sperm harvested 14 days post Dex and vehicle injection. Sheet 3: Raw values of qRT-PCR analysis for caput and cauda sperm circRNAs.
