# Supplementary material for: Single paternal dexamethasone challenge programs offspring metabolism and reveals multiple candidates in RNA-mediated inheritance
Source: iScience. 2021 Jul 16;24(8):102870. doi: 10.1016/j.isci.2021.102870 (PMC8346661; doi:10.1016/j.isci.2021.102870)
Supplement: Document S1. Figures S1–S6 [file mmc1.pdf]

iScience, Volume *24*

Supplemental information

Single paternal dexamethasone challenge programs offspring metabolism and reveals multiple candidates in RNA-mediated inheritance

Katharina Gapp, Guillermo E. Parada, Fridolin Gross, Alberto Corcoba, Jasmine Kaur, Evelyn Grau, Martin Hemberg, Johannes Bohacek, and Eric A. Miska

Supplementary Information

Supplementary Figure 1 related to Figure 1 and 4

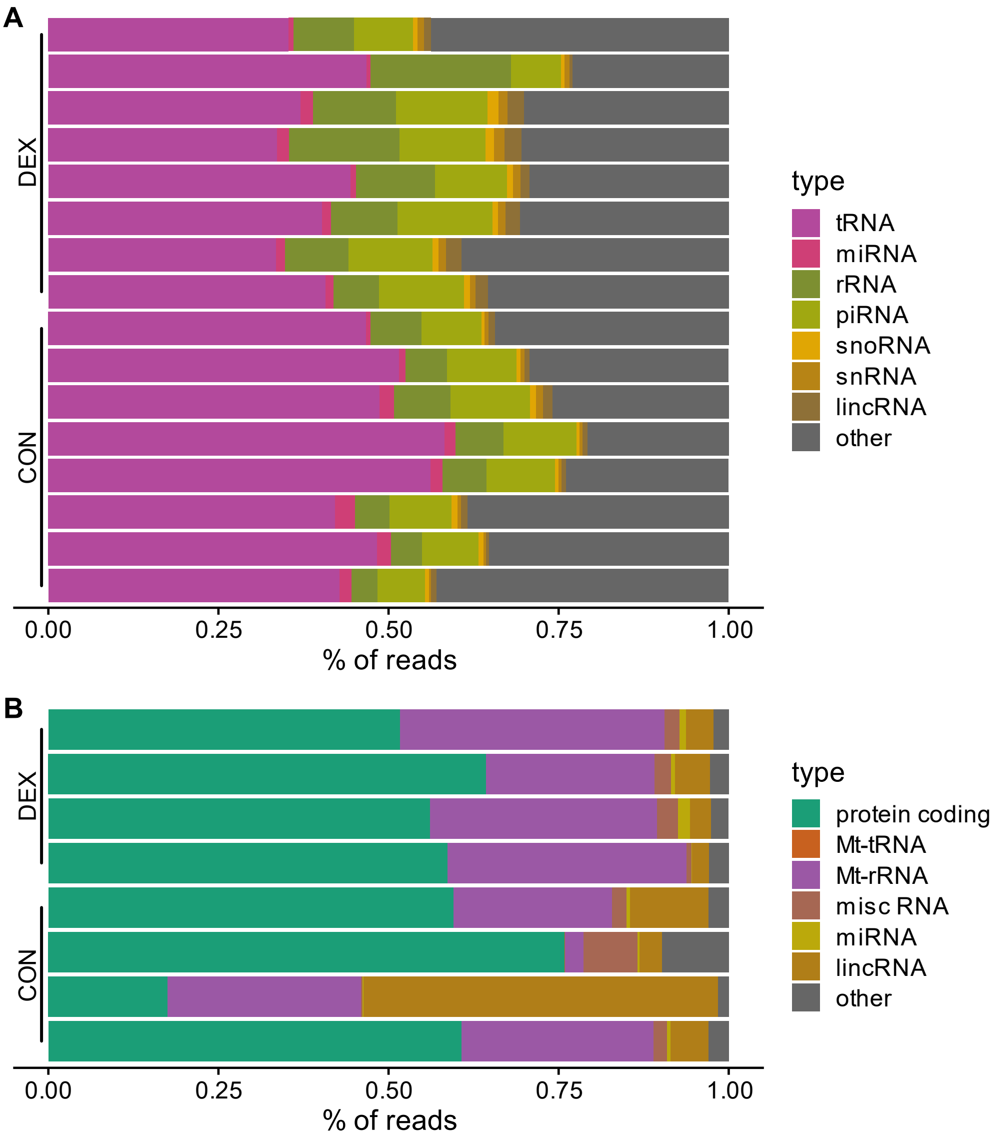

Relative percentage of RNA Ensemble biotypes and tRNA matching reads (A) in small RNA sequencing libraries of 8 sperm samples from control males and 8 sperm samples 14 days post dexamethasone injection. (B) long RNA sequencing libraries of 4 sperm samples from control males and 4 sperm samples 14 days post dexamethasone injection.

Supplementary Figure 2 related to Figure 2

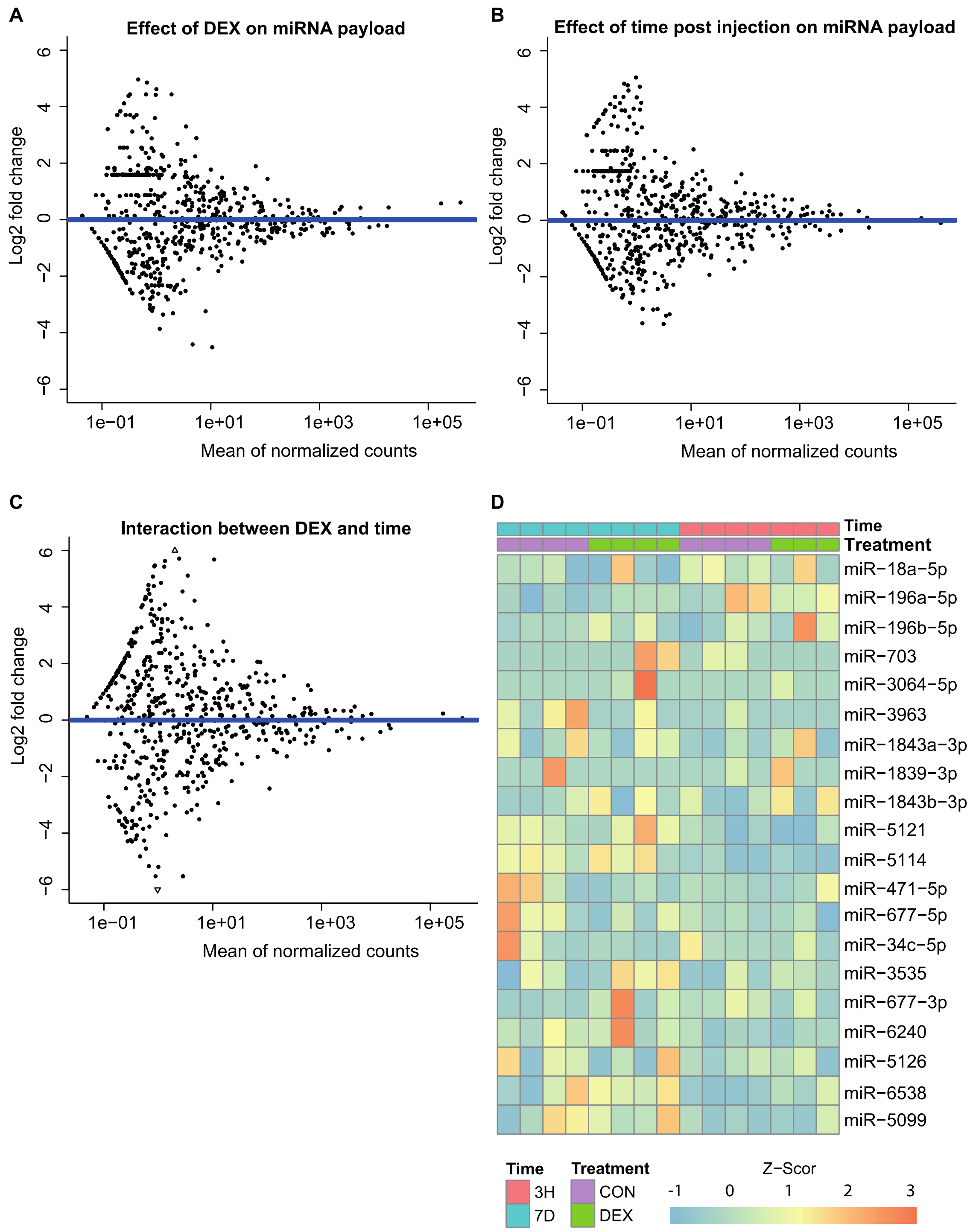

Mature sperm miRNA payload as determined by next generation sequencing. Deseq2 analysis of sperm samples collected 3 hours and 14 days post dexamethasone injection did not reveal any significant effects of (A) treatment (dexamethasone injection), (B) time (time elapsed since injection) nor an (C) interaction between the two. (D) Heat map depicting those miRNAs that are significantly affected in the data obtained from 14 days post injection, at 3 hours and 7 days post dexamethasone injection.

Supplementary Figure 3 related to Figure 3

A B

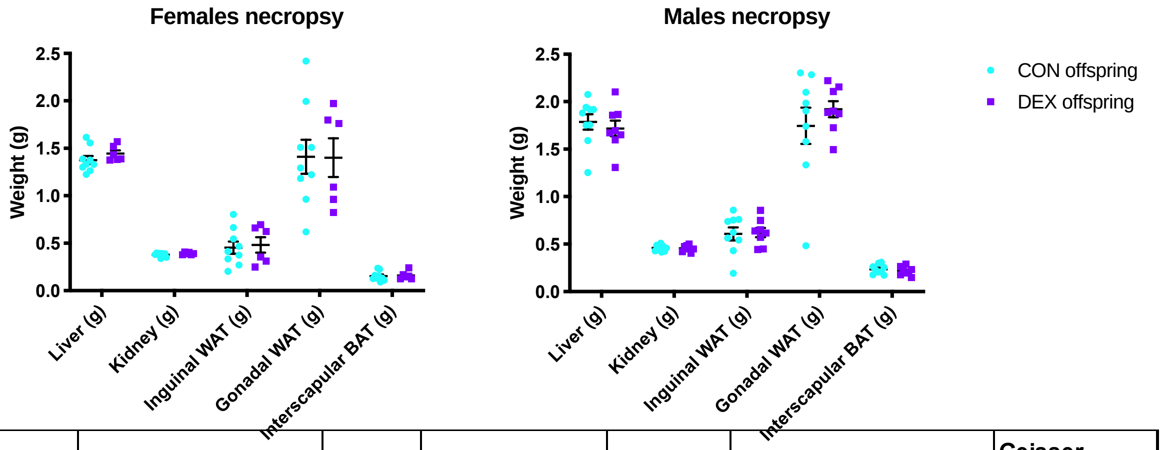

C

Variable	Fixed effects (type III)	P value	P value summary	Statistically	F (DFn, DFd)	Geisser-Greenhouse's epsilon
BMI	time	<0.0001	****	Yes	F (2.087, 144.7) = 41.99	0.6957
BMI	sex	<0.0001	****	Yes	F (1, 71) = 76.55	
BMI	treatment	<0.0001	****	Yes	F (1, 71) = 49.86	
BMI	time x sex	<0.0001	****	Yes	F (3, 208) = 33.75	
BMI	time x treatment	0.0008	***	Yes	F (3, 208) = 5.834	
BMI	sex x treatment	0.2181	ns	No	F (1, 71) = 1.544	
BMI	time x sex x treatment	0.4323	ns	No	F (3, 208) = 0.9196	

	ANOVA table	SS	DF	MS	F (DFn, DFd)	P value
GTT	time	60.74	4	15.18	F (3.314, 122.6) = 23.85	P<0.0001
GTT	sex	732.6	1	732.6	F (1, 37) = 162.6	P<0.0001
GTT	treatment	9.901	1	9.901	F (1, 37) = 2.198	P=0.1467
GTT	time x sex	31.81	4	7.951	F (4, 148) = 12.49	P<0.0001
GTT	time x treatment	13.7	4	3.425	F (4, 148) = 5.380	P=0.0005
GTT	sex x treatment	3.936	1	3.936	F (1, 37) = 0.8737	P=0.3560
GTT	time x sex x treatment	13.73	4	3.432	F (4, 148) = 5.392	P=0.0004
GTT	Subject	166.7	37	4.504		
	Residual	94.21	148	0.6366		
ITT	time	60.74	4	15.18	F (3.314, 122.6) = 23.85	P<0.0001
ITT	sex	732.6	1	732.6	F (1, 37) = 162.6	P<0.0001
ITT	treatment	9.901	1	9.901	F (1, 37) = 2.198	P=0.1467
ITT	time x sex	31.81	4	7.951	F (4, 148) = 12.49	P<0.0001
ITT	time x treatment	13.7	4	3.425	F (4, 148) = 5.380	P=0.0005
ITT	sex x treatment	3.936	1	3.936	F (1, 37) = 0.8737	P=0.3560
ITT	time x sex x treatment	13.73	4	3.432	F (4, 148) = 5.392	P=0.0004
ITT	Subject	166.7	37	4.504		
	Residual	94.21	148	0.6366		

Tissue	P value	Difference	SE of difference	t ratio
Liver (g)	0.27665	-0.06789	0.05979	1.136
Kidney (g)	0.188702	-0.01267	0.009131	1.387
Inguinal WAT (g)	0.776367	-0.02972	0.1025	0.29
Gonadal WAT (g)	0.968101	0.01122	0.2753	0.04077
Interscapular BAT (g)	0.801259	-0.006444	0.02508	0.2569
Liver (g)	0.562456	0.06872	0.116	0.5923
Kidney (g)	0.788758	0.004292	0.01573	0.2728
Inguinal WAT (g)	0.872972	-0.014	0.08608	0.1626
Gonadal WAT (g)	0.434173	-0.1747	0.2174	0.8036
Interscapular BAT (g)	0.432084	0.01892	0.02343	0.8073

Necropsy followed by weighing of dissected tissue from offspring of control injected and Dexamethasone injected males. Comparison of the weight of liver, kidney, inguinal and gonadal white adipose tissue (WAT) as well as interscapular brown adipose tissue (BAT) did not reveal significant differences between (A) female and (B) male offspring of control and dexamethasone injected males. (C) Statistical values obtained by overall ANOVAs (BMI, GTT, ITT) and Multiple t-tests corrected for multiple comparisons (necropsy). All Data besides male gonadal WAT showed equal distribution of variances. Graphs show scattered dot plots with standard error of the mean. ITT = insulin tolerance test, GTT= Glucose tolerance test, BMI= Body mass index, WAT = white adipose tissue. BAT= brown adipose tissue.

Supplementary Figure 4 related to Figure 5

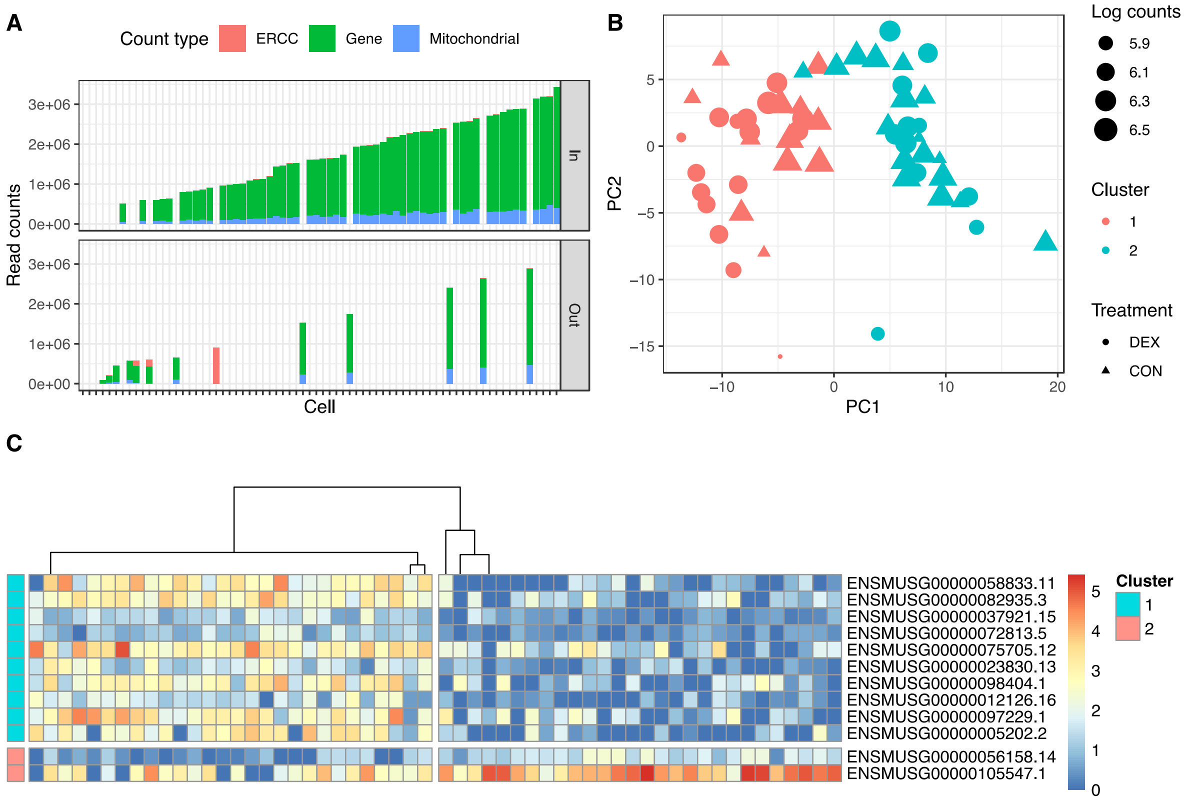

Single- 2-cell embryo sequencing using the Smartseq method. (A) Result of the quality filters implemented to select the 2 cell embryos that were used for downstream analyses. Libraries (embryos/cells) that contained a suboptimal number of mitochondrial (>15%) or ERCC mapping reads (>10%) or yielded less than 500 000 reads were excluded (displayed in Out). Most libraries showed a high number of gene mapping reads and were retained and processed for further analysis (displayed in In). (B) PCA results shown as a single panel, revealing segregation of embryos for the first two principle components (matching almost perfectly cluster 1 (red) and cluster 2(blue)) and additionally depicting read count number (size of circle) and assignment to treatments (offspring embryos of control fathers = CON, dots and fathers who were injected with dexamethasone 14 days prior sperm harvest = DEX, triangles). PC2 is attributed to technical factors such as read counts. (C) Marker genes identified by sc3 for C1 and C2 clusters.

Supplementary Figure 5 related to Figure 5 and 6

circAltas ID	microRNA name	#binding sites of miRanda	#bingding sites of targetScan
mmu-Dennd1b_0017	mmu-miR-201-3p	1	1
mmu-Dennd1b_0017	**mmu-miR-3110-5p**	1	1
mmu-Dennd1b_0017	mmu-miR-1953	1	1
mmu-Dennd1b_0017	mmu-miR-1981-5p	1	1
mmu-Dennd1b_0017	**mmu-miR-706**	1	1
mmu-Dennd1b_0017	mmu-miR-6363	1	1
mmu-Dennd1b_0017	mmu-miR-3074-5p	1	1
mmu-Dennd1b_0017	**mmu-miR-1955-5p**	1	2
mmu-Dennd1b_0017	mmu-miR-675-3p	1	1
mmu-Dennd1b_0017	mmu-miR-343	1	2
mmu-Dennd1b_0017	mmu-miR-3083-5p	1	1
mmu-Dennd1b_0017	mmu-miR-3073a-5p	1	1
mmu-Dennd1b_0017	mmu-miR-9-5p	1	1
mmu-Dennd1b_0017	mmu-miR-378b	1	1
mmu-Tasp1_0039	mmu-miR-221-5p	1	1
mmu-Tasp1_0039	mmu-miR-5627-3p	1	3
mmu-Tasp1_0039	mmu-miR-298-5p	1	3
mmu-Tasp1_0039	mmu-miR-3058-5p	1	1
mmu-Tasp1_0039	mmu-miR-207	1	3
mmu-Tasp1_0039	mmu-miR-709	1	1
mmu-Tasp1_0039	mmu-miR-6537-3p	1	1
mmu-Tasp1_0039	mmu-miR-713	1	2
mmu-Tasp1_0039	mmu-miR-3094-3p	1	2
mmu-Tasp1_0039	mmu-miR-100-3p	1	2
mmu-Tasp1_0039	mmu-miR-1906	1	3
mmu-Tasp1_0039	mmu-miR-320-5p	1	3
mmu-Tasp1_0039	mmu-miR-3082-3p	1	2
mmu-Tasp1_0039	**mmu-miR-706**	1	1
mmu-Tasp1_0039	**mmu-miR-3110-5p**	1	1
mmu-Tasp1_0039	mmu-miR-6900-5p	1	1
mmu-Tasp1_0039	**mmu-miR-1955-5p**	1	1
mmu-Tasp1_0039	mmu-miR-674-5p	1	1
mmu-Tasp1_0039	mmu-miR-351-3p	1	1
mmu-Tasp1_0039	mmu-miR-5104	1	1
mmu-Tasp1_0039	mmu-miR-504-3p	1	1
mmu-Tasp1_0039	mmu-miR-152-5p	1	1
mmu-Tasp1_0039	mmu-miR-1897-5p	1	1
mmu-Tasp1_0039	mmu-miR-3105-5p	1	1
mmu-Tasp1_0039	mmu-miR-742-3p	1	2
mmu-Tasp1_0039	mmu-miR-1969	1	2
mmu-Tasp1_0039	mmu-miR-1960	1	2
mmu-Tasp1_0039	mmu-miR-24-3p	1	2
mmu-Tasp1_0039	mmu-miR-188-5p	1	1

CircRNA targets as predicted by CircAtlas. Each column displays results from miRanda and targetScan.

miRNAs that are targeted by both circRNAs are highlighted in purple.

Supplementary Figure 6 related to Figure 1

A

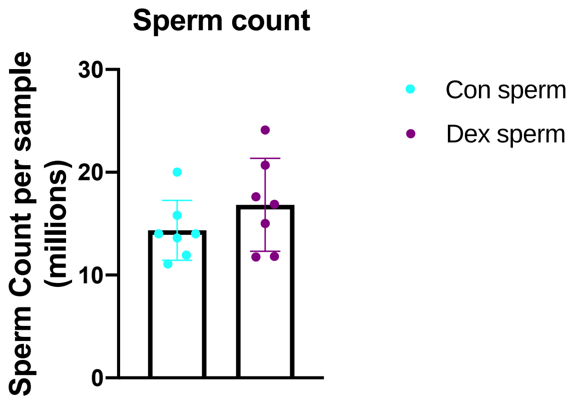

B

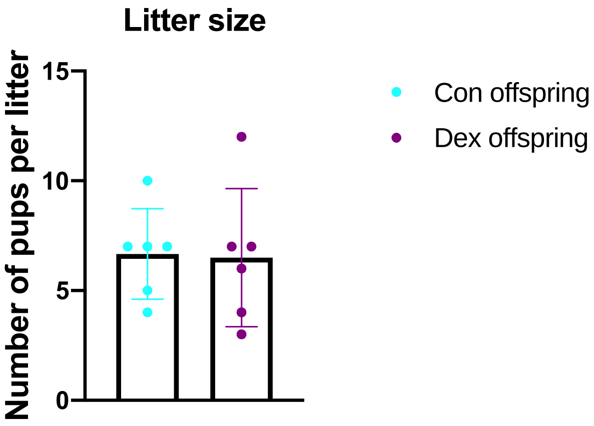

C

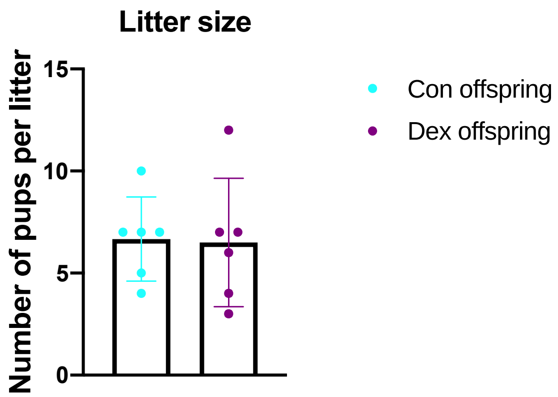

Fertility measures following Dexamethasone treatment. (A) Sperm cell count 14 days post vehicle (n=7) or Dex (n=7) injection did not reveal any difference in sperm number (t(12)=1.221, p=0.25) suggesting no impairing effect of Dex on spermatogenesis. (B) Count of fertilized oocytes (as of the appearance of the second pronucleus) over total available oocytes of 6 replicates of cryopreserved sperm from a pool of 2 dex injected versus 2 vehicle injected males did reveal no significant difference in the fertilization rate between dex and vehicle sperm. (C) Number of pups per litter was similar in offspring resulting from in vitro fertilization with sperm from fathers that were injected with Vehicle (n=6) or Dex (n=6) (t(12)=1.122, p=0.25).
